# Evaluation of Nitrobenzyl Derivatives of Camptothecin as Anti-Cancer Agents and Potential Hypoxia Targeting Prodrugs

**DOI:** 10.3390/molecules23082041

**Published:** 2018-08-15

**Authors:** Dinghua Liang, Xing Wu, Brian B. Hasinoff, David E. Herbert, Geoffrey K. Tranmer

**Affiliations:** 1 College of Pharmacy, Faculty of Health Science, University of Manitoba, Winnipeg, MB R3E 0T5, Canada; liangd@myumanitoba.ca (D.L.); xingwu@hotmail.com (X.W.); b.hasinoff@gmail.com (B.B.H.); 2 Department of Chemistry, Faculty of Science, University of Manitoba, Winnipeg, MB R3T 2N2, Canada; david.herbert@umanitoba.ca

**Keywords:** camptothecin, hypoxia-activated prodrug, topoisomerase I inhibitor, K562 cells, anti-cancer agents, SN-38

## Abstract

As part of our initial efforts into developing a tumor-targeting therapy, C-10 substituted derivatives of a camptothecin analog (SN-38) have been synthesized (2-, 3- and 4-nitrobenzyl) for use as potential hypoxia-activated prodrugs and evaluated for their cytotoxicity, topoisomerase I inhibition and electrochemical (reductive) properties. All three derivatives were found to possess reduced toxicity towards human leukemia K562 cells compared to SN-38, validating a condition for prodrug action. Using an MTS assay, IC_50_’s were found to be 3.0, 25.9, 12.2 and 58.0 nM for SN-38, 2-nitro-, 3-nitro- and 4-nitrobenzyl-C_10_-substituted-SN-38, respectively, representing an 8-, 4- and 19-fold decrease in cytotoxicity. Using a topoisomerase I assay, one of the analogs (4-nitrobenzyl) was shown to inhibit the ability of this enzyme to relax supercoiled pBR322 DNA, at a similar concentration to the clinically-approved active metabolite SN-38. Cyclic voltammetry detailed the reductive nature of the analogs, and was used to infer the potential of these compounds to serve as hypoxia-targeting prodrugs. The electrochemical results also validated the quasi-reversible nature of the first reduction step, and served as a proof-of-principle that hypoxia-targeting prodrugs of SN-38 can participate in a redox-futile cycle, the proposed mechanism of activation and targeting. Chemical reduction of the 4-nitrobenzyl analog led to the formation/release of SN-38 and validated the prodrug ability of the C-10 substituted derivative.

## 1. Introduction

Camptothecins are a family of compounds that are structurally related to the natural product camptothecin (CPT), which was initially extracted from a tree native to southern China, *Camptotheca acuminata*. Several of these compounds have entered into clinical trials for their anti-cancer properties and their ability to inhibit topoisomerase I, with both topotecan and irinotecan receiving FDA approval in 1996 [1]. As part of our research program on the development of targeted anti-cancer agents, we set out to evaluate the ability of nitrobenzyl derivatives of camptothecins to serve as: (a) anti-cancer agents; (b) inhibitors of topoisomerase I; and (c) hypoxia-activated prodrugs. Initially, following the synthesis of a series of nitrobenzyl derivatives of camptothecin, we evaluated their ability to serve as anti-cancer agents using K562 cells and as inhibitors of topoisomerase I using an enzymatic assay. Additionally, the prodrug ability of our derivatives was also studied as nitrobenzyl functional groups have been previously used as part of a strategy to target hypoxia in tumors (*vide infra*).

Hypoxia in cells is characterized by an oxygen-deficient state that results in a functional change of cellular processes [2]. In solid tumors, a state of hypoxia can exist as a result of inadequate oxygen supply caused by abnormal vasculature or irregular blood perfusion [3]. As a result of the decrease in oxygen concentration, the metastatic aggressiveness, multi-therapeutic resistance and inferior prognosis of hypoxic tumor microenvironments have been widely acknowledged and studied [4,5]. In particular, the oxygenation state of tumors has been shown to be the strongest independent prognostic factor, at least for cervical cancer [6]. Due to these drawbacks, there is an urgent need to develop therapeutic strategies that specifically target hypoxia in tumors. In order to address this issue, and as part of our initial efforts into this field, we evaluated the reduction potential of nitrobenzyl derivatives of SN-38 in order to infer their ability to target hypoxic environments, in addition to their anti-cancer properties and topoisomerase I inhibitory activity.

Hypoxia-activated prodrugs (HAP) are stable analogs of therapeutic agents under normal oxygen conditions, but become activated in oxygen deficient cells, generating (or releasing) cytotoxins within (or near) the tumor [7]. The most extensively explored strategy for activating prodrugs under hypoxic conditions is to utilize the chemically reducing environment that persists within hypoxic tumor tissues [8,9]. In general, this approach involves the incorporation of a functional group within the HAP that is selectively reduced within hypoxic environments leading to the generation or release of a cytotoxic agent. In this manner, hypoxia can be utilized for its bioreductive environment, selectively concentrating the cytotoxic agent within hypoxic tumors, and serving as a cancer targeting strategy. Nitroaromatic compounds are one of the major classes of bioreductive functional groups that can serve this purpose. Several HAP that incorporate either a 5- or 6-membered nitroaromatic (Ar-NO_2_) have entered into various stages of clinical trials, such as PR-104 or TH-302 [10]. In each of these cases, a nitroaromatic moiety (the trigger) is attached to a cytotoxic agent, the effector [11]. Under hypoxic conditions, the strongly electron-withdrawing nitro group (-NO_2_) is reduced by reductases to an electron-donating group, such as an amine (-NH_2_) or hydroxylamine (-NHOH), resulting in a dramatic change in the electron density of the aromatic group. The elevation in electron density results in the activation or release of the effector, generating an anti-cancer agent within the hypoxic tumor. The overall activation process is called an ‘electronic switch’ [12,13] and aptly serves to localize cytotoxins to hypoxic regions. For more information on the development of HAP, the prodrug activation process and reductases, several reviews have been produced [7,14,15].

## 2. Results

**Figure 1:**
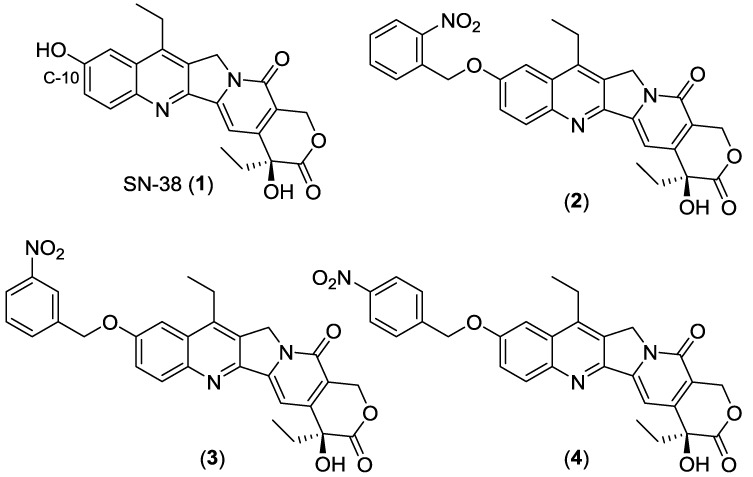
SN-38 (**1**) and potential hypoxia activated prodrugs **2**, **3** and **4**.

It is our plan to generate novel anti-cancer agents by synthesizing derivatives of camptothecins and evaluating their anti-cancer properties, with the prospect of developing these into hypoxia-targeting agents. SN-38, the active metabolite of irinotecan, was selected as our parent template from which we could easily generate a series of analogs that we hypothesize could serve as stand-alone anti-cancer agents. Additionally, we chose to generate nitrobenzyl derivatives of camptothecins as these compounds may also possess the additional benefit of targeting hypoxic tumor environments. To the best of our knowledge, nitrobenzyl analogs of SN-38 (**1**) have not appeared in the literature, nor have been studied as HAP’s. To accomplish this task, we attached nitrobenzyl groups (the trigger) to the C-10 position of SN-38 (the effector), as we hypothesize that nitroaromatic analogs of camptothecin may have the ability to serve as hypoxia-targeting anti-cancer agents. However, we first needed to establish whether this class of compounds can serve as stand-alone anti-cancer agents, followed by their evaluation as inhibitors of topoisomerase I, and finally, evaluation of the electrochemical reduction potentials of the compounds will allow us to ascertain their potential to serve as hypoxia-activated prodrugs. Ideally, to serve as an HAP, rather than as a stand-alone anti-cancer agent, the compounds should be less cytotoxic than the parent compound and capable of being reduced under hypoxic cellular conditions. To this end, we synthesized three nitrobenzyl derivatives of camptothecin, C-10—2-nitro (**2**), 3-nitro (**3**) and 4-nitrobenzyl (**4**), as shown in Figure 1. We then evaluated the cytotoxic properties of these three compounds against human leukemia K562 cells and examined their effects on inhibiting the ability of topoisomerase I to relax pBR322 DNA. In the final stage of our study, we determined the electrochemical properties of the nitroaromatics using cyclic voltammetry as a measure of their potential to serve as HAP. 

### 2.1. Synthesis of Analogs ***2***, ***3*** and ***4***

**Scheme 1:**
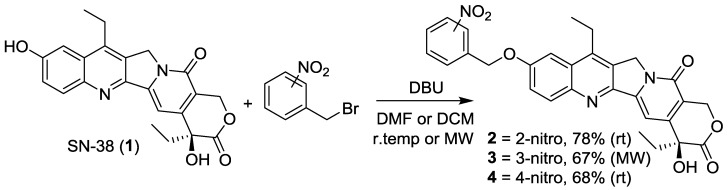
Synthesis of nitrobenzyl analogs via alkylation reaction.

Irinotecan is the clinically approved prodrug of SN-38 that possesses a bipiperidine-carboxylate group attached to a C-10 hydroxyl functionality of the camptothecin core. Experience in prescribing and clinically using this drug has accumulated for 20 years, with abundant clinical data showing that it is generally safe when used as a chemotherapeutic agent. As a result of the clinical data available, a strong rationale exists that predicts that SN-38 could serve as a useful template for further drug development. Derivatives may possess the ability to serve as potential hypoxia-activated prodrugs and retain a high likelihood of maintaining clinical effects. For the design of HAP analogs of SN-38, a fragmentation-activation strategy is preferred, wherein the prodrug possesses reduced cytotoxicity, and is metabolically activated in vivo, releasing the clinically-approved active metabolite SN-38, as highlighted by a nitrobenzyl (trigger)—SN-38 (effector) strategy. For example, the installation of a bipiperidine-carboxylate group at the C-10 position of SN-38 (as with irinotecan) makes the prodrug 1000-fold less active than its unsubstituted metabolite SN-38 [1]. In an analogous fashion, modification of the C-10 position of SN-38 with functional groups that are capable of metabolic activation under hypoxic environments, such as nitroaromatics, should lead to less-active chemotherapeutic agents and provide for prodrug ability. To this end, three different nitroaromatic groups (2-nitro, 3-nitro and 4-nitrobenzyl) were selected as appropriate triggers for attachment to SN-38 at the C-10 position, in order to generate a series of potential HAP’s. A variety of alkylating reactions were investigated in order to provide access to the nitrobenzyl analogs of SN-38, including using mesylates and bromides as leaving groups. Alternately, Mitsunobu conditions were also explored to give access to the desired products from nitrobenzyl alcohols. After extensive study, it was deduced that using nitrobenzyl bromides under alkylating conditions provided for the most consistent route to the desired products. At room temperature, both 2-nitrobenzyl bromide and 4-nitrobenzyl bromide provided for access to the 2-nitro and 4-nitrobenzyl derivatives of SN-38, in 78% and 68% isolated yields, respectively, compounds **2** and **4**, as shown in Scheme 1. In general, 2 equivalence of the benzyl bromide was used, along with 2.2 equiv. of diazabicyclo [5.4.0] undec-7-ene (DBU) in dimethylformamide (DMF). 3-Nitrobenzyl bromide was found to be much more sluggish under these conditions, with reactions generally requiring very long reaction times and resulting in poor isolated yields. An alternate method, however, was developed that used microwave irradiation to provide for the 3-nitrobenzyl-SN-38 analog (**3**) in 67% isolated yield, as shown in Scheme 1. For this particular reaction, 3 equiv. of 3-nitrobenzyl bromide was used, along with 4 equiv. of DBU in dry dicloromethane (DCM), with 10 min of microwave irradiation at 66 °C using the PowerMax mode (simultaneous air cooling). Overall, alkylation reactions were developed that provided for access to the desired analogs of SN-38 in good yields using a mild room temperature reaction, or under microwave conditions, using DBU as base, which were more than sufficient for the purposes of development of HAP analogs of SN-38.

### 2.2. Cell Viability Assay

**Figure 2:**
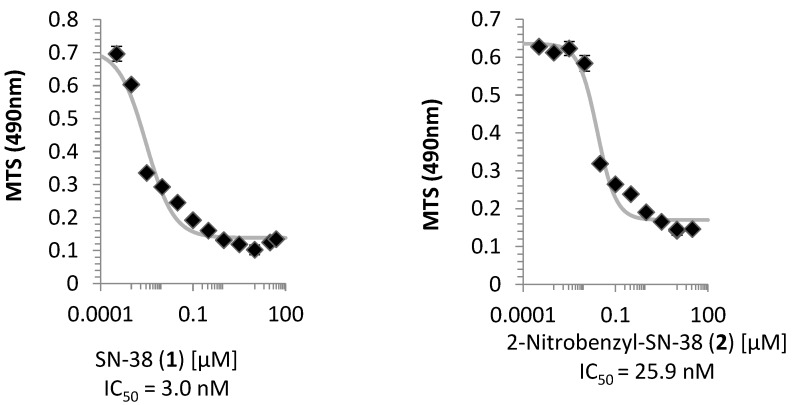

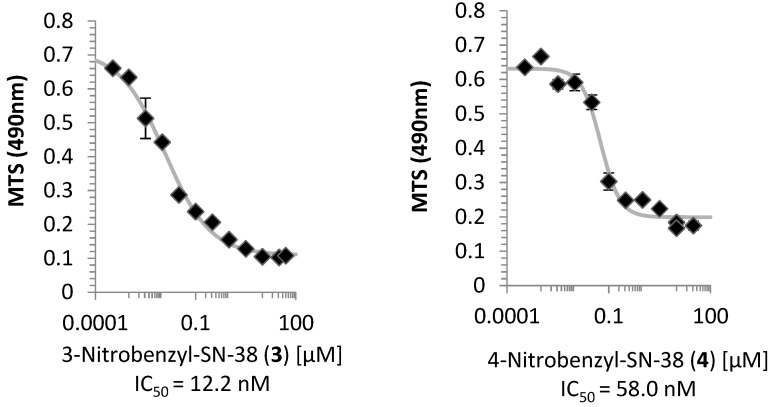
Dose response curves and IC_50_ values for SN-38 (**1**); 2-nitrobenzyl-SN-38 (**2**); 3-nitrobenzyl-SN-38 (**3**) and 4-nitrobenzyl-SN-38 using an MTS assay.

The three nitrobenzyl derivatives (**2**, **3**, **4**) and SN-38 were evaluated on their ability to attenuate cell growth using human leukemia K562 cells. Cells harvested during exponential growth were seeded with approximately 1150 cells per well in 96-well microtiter plates. The potential prodrugs were dissolved in dimethyl sulfoxide (DMSO) and tested with 11 different concentrations to construct a dose-response curve, with a final volume of 200 µL per well. Each analog was tested using two adjacent lanes of wells, with the final concentration not exceeding 0.5% (*v*/*v*) of DMSO, and an amount of drug that possessed no detectable effect on K562 cell growth. The cells were incubated for 48 h and assayed using standard MTS conditions. The values for the half maximal inhibitory concentration (IC_50_) were calculated by fitting the average absorbance-concentration data of two lanes to a four-parameter logistic equation. The curves for each compound (**1**–**4**) can be found in Figure 2, with the IC_50_’s of SN-38, and 2-nitro, 3-nitro and 4-nitrobenzyl found to be 3.0, 25.9, 12.2 and 58 nM, respectively. As expected, all nitrobenzyl analogs were found to be less toxic to human K562 cells than SN-38 with a magnitude of approximately 8-, 4- and 19-fold, respectively.

### 2.3. Topoisomerase I Inhibition Assay

**Figure 3:**
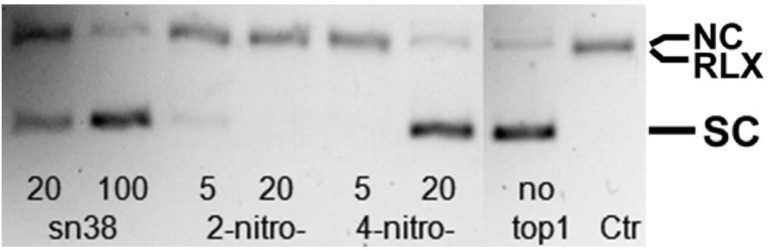
Effects of SN-38 (**1**), 2-nitrobenzyl-SN-38 (**2**) and 4-nitrobenzyl-SN-38 (**4**) on their ability to inhibit the topoisomerase I-induced relaxation of pBR322 DNA. NC, nicked circular DNA; RLX, relaxed DNA; SC, supercoiled DNA; “sn38”, “2-nitro-” and “4-nitro-” denote the tested drugs SN-38 (**1**), 2-nitrobenzyl-SN-38 (**2**) and 4-nitrobenzyl-SN-38 (**4**), respectively; numbers indicate the final concentration [µM] of the tested drug in the assay mixture applied to the lanes above; all lanes except the lane “no top1” contained topoisomerase 1; no drug was added to the lane “Ctr”.

From the initial cell viability assay results, both the 2-nitrobenzyl- and 4-nitrobenzyl-SN-38 analogs appeared to be much less cytotoxic than SN-38 itself, with 8- and 19-fold differences, respectively. One of the principle aspects of a hypoxia-activated prodrug is the need to have the parent prodrug compound be significantly less potent than the active metabolite. As a result, the inhibitory effects of SN-38, 2-nitrobenzyl- and 4-nitrobenzyl-SN-38 on the ability of topoisomerase I to relax pBR322 DNA were examined and shown on the color-reversed fluorescent image of a gel stained with ethidium bromide, as shown in Figure 3. Using this method, the gel highlights the supercoiled DNA (SC) which is located well below the relaxed DNA (RLX) and nicked circular DNA (NC). With the exception of the “no top1” lane, all lanes contained topoisomerase I, and the “Ctr” lane contained no drug. In principle, the lane containing no drug should highlight relaxed DNA, via the action of topoisomerase I, while the lane that does not contain topoisomerase I should contain mostly supercoiled (unrelaxed) DNA. The known topoisomerase I inhibitor SN-38 was found to inhibit the DNA-supercoil-relaxing activity of the enzyme at two concentrations (20 and 100 µM) in a dose-dependent manner. For the 2-nitrobenzyl analog, it did not show inhibitory effects at two concentrations (5 and 20 µM), while 4-nitrobenzyl-SN-38 did show an inhibitory effect at the higher concentration of 20 µM, but not at the lower concentration of 5 µM.

### 2.4. Cyclic Voltammetry

**Figure 4:**
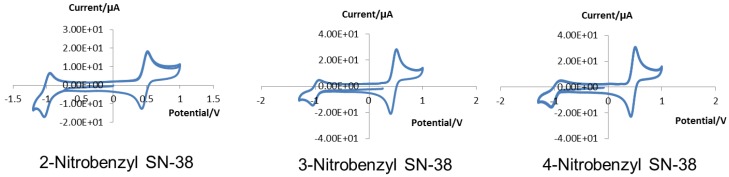
Cyclic voltammograms of 2-nitrobenzyl (**2**); 3-nitrobenzyl (**3**) and 4-nitrobenzyl (**4**) analogs.

As a measure of the intrinsic potential required to reduce the nitroaromatic functionality of the candidate HAP’s, the electrochemical properties of 2-, 3- and 4-nitrobenzyl analogs of SN-38 were measured using cyclic voltammetry. Experiments were performed using a CHI760C electrochemical workstation with a three-electrode cell (platinum wire as auxiliary electrode, Ag/Ag^+^ as reference electrode, and a glassy carbon disc as working electrode). All potentials were referenced to ferrocene/ferrocenium [16] as an internal standard. Experiments were performed using 1 mM solutions of the SN-38 derivatives in DMSO containing 0.1 M lithium perchlorate as a supporting electrolyte. The solutions were deoxygenated by bubbling argon through the solutions for >10 min prior to measurements. Table 1 highlights the important parameters for the collected cyclic voltammograms (Figure 4), wherein E_pa_^Ag^(E_pc_^Ag^) is the anodic (cathodic) peak potential for the nitrobenzyl SN-38 analogs against Ag/Ag^+^ electrode; i_pa_(i_pc_) is the anodic (cathodic) peak current; *i*_pa_/*i*_pc_ is the peak current ratio; and E_pa_^Ag^(Fc)[E_pc_^Ag^(Fc)] is the anodic (cathodic) peak potential of the ferrocene internal standard against the Ag/Ag^+^ electrode.

The half-wave potential (*E*_1/2_) (also known as formal reduction potential, *E*^o’^ or *E*_f_) is:

(1) $$E_{1/2}=\frac{E_{\mathrm{pa}}+E_{\mathrm{pc}}}{2}$$

*E*_1/2_^Ag^ [*E*_1/2_^Ag^(Fc)] is the half-wave potential of SN-38 derivatives [ferrocene] against Ag/Ag^+^ electrode. *E*_1/2_^Fc^ is the half-wave potential of SN-38 derivatives against the internal standard ferrocene/ferrocenium (Fc/Fc^+^) redox couple:

(2) $${E_{1/2}}^{\mathrm{Fc}}={E_{1/2}}^{\mathrm{Ag}}-{E_{1/2}}^{\mathrm{Ag}}\left(\mathrm{Fc}\right)$$

*E*_1/2_^NHE^ is the value against normal hydrogen electrode (NHE) for the convenience of comparison with literature data, which is converted via:

(3) $${E_{1/2}}^{\mathrm{NHE}}={E_{1/2}}^{\mathrm{Fc}}+450\;\mathrm{mV}+250\;\mathrm{mV}$$

where the +450 mV [16] is to relate a potential to saturated calomel electrode (SCE) and the +250 mV [17] is to convert a value against SCE to a value against NHE. Note: *E*_1/2_ (450 mV [16]) of ferrocene in DMSO with 0.1 M tetrabutylammonium perchlorate as supporting electrolyte vs. SCE (*E*_1/2_^Fc^ DMSO/[nBu_4_N][ClO_4_]) was used for the conversion of *E*_1/2_^Fc^ DMSO/LiClO_4_ (1 M lithium perchlorate as supporting electrolyte). For the same reason, the conversion constant between SCE and NHE in acetonitrile (250 mV [17]) was used for converting data acquired in DMSO.

### 2.5. Chemical Reduction of Prodrug

**Scheme 2:**
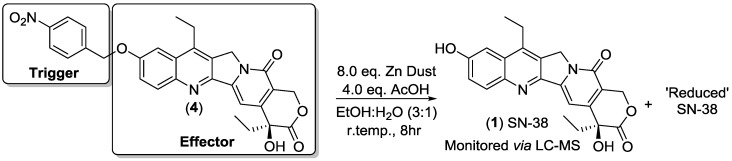
Chemical reduction of 4-nitro-prodrug and release of SN-38 and the formation of a reduced form of SN-38 by zinc dust and acetic acid (monitored via LC-MS).

We set out to provide further evidence that our nitroaromatic analogs (**2**, **3**, **4**) can serve as prodrugs via reduction of the nitro groups (trigger) and subsequent release of SN-38 (effector). There are a number of relatively mild chemical means that can provide for the selective reduction of nitroaromatic groups to the corresponding amines [18,19,20]. We found that using zinc dust and acetic acid provided an effective means to chemically reduce the nitroaromatic group of the 4-nitro-prodrug (**4**) and led to the formation of SN-38 and a reduced form of SN-38, as monitored by LC-MS (see **Supplementary Materials**). Initially, the reaction solvents were purged of any oxygen using argon, and the reaction was performed under inert conditions (argon). After 10 min of reaction time, an aliquot of the crude reaction mixture was prepared for LC-MS and indicated the formation of both SN-38 (393 [M + H]^+^) and a reduced form of SN-38 (395 [M + H]^+^), as denoted by a mass of +2H. After 8 h of reaction time, an additional aliquot of the crude reaction mixture indicated further formation of SN-38, as indicated by LC-MS, shown in Scheme 2, as well as unreacted starting material, and a reduced form of SN-38.

## 3. Discussion

### 3.1. Cell Viability and Topoisomerase I Inhibition Assay

Considering the cell viability assay with human leukemia K562 cells, the three nitrobenzyl derivatives of SN-38 were found to possess less cytotoxicity than the parent compound. Based on the principal of prodrug design which requires that the derivative analogs should show reduced toxicity, we can validate the premise that our compounds have the potential to serve as prodrugs. This result also supports the consideration/hypothesis that a bulky trigger installed to the C-10 hydroxyl functionality of SN-38 reduces the anticancer activity of the clinically approved metabolite. The results also indicate that the most effective deactivating trigger was found to be the 4-nitrobenzyl derivative, and possessed a 19-fold reduction in cytotoxicity during incubation with K562 cells compared to SN-38. The 3-nitrobenzyl derivative was found to only have a nominal decrease in cytotoxicity of 4-fold, while the 2-nitrobenzyl compound had an 8-fold decrease in cytotoxicity. As part of future work, it is proposed that bulkier triggers would need to be designed to decrease the cytotoxicity to cancer cells even further, and lead to more effective prodrug design.

For the two analogs which showed the greatest decrease in cytotoxicity, these two compounds were tested using a topoisomerase I inhibitory assay which indicated the ability of compounds to inhibit the ability of the enzyme to relax supercoiled DNA. For these two analogs, 2-nitrobenzyl and 4-nitrobenzyl, only the latter compound was found to inhibit the topoisomerase I-induced relaxation of pBR322 DNA. This is an interesting observation when compared to the cell viability assay results, as the more cytotoxic derivative was not found to inhibit topoisomerase I activity at the highest concentration. The 2-nitrobenzyl analog did not show any inhibitory effects on topoisomerase I function at a concentration of 20 µM, however, this compound appeared to be more toxic to K562 cells than the 4-nitrobenzyl analog which was found to be effective in inhibiting topoisomerase I in our assay at 20 µM concentration. The IC_50_’s for 2- and 4-nitrobenzyl-SN-38 were found to be 25.9 nM and 58.0 nM, respectively. The simplest explanation for this result is that the 2-nitrobenzyl derivative may operate via a topoisomerase I inhibition-independent cytotoxic mechanism, such as HIF-1 inhibition. What is clear is that the 4-nitrobenzyl derivative appeared to inhibit topoisomerase I activity at a similar concentration as SN-38 (20 µM), but was found to be nearly 20-fold less cytotoxic to K562, validating the premise that the compounds developed herein are capable of serving as anticancer agents, and inhibiting topoisomerase I.

### 3.2. Cyclic Voltammetry

In order to ascertain whether our compounds could serve as hypoxia activated prodrugs, we used cyclic voltammetry as a surrogate measure to evaluate the electrochemical properties of our analogs, and infer the relative ability of these compounds to serve as a prodrug under hypoxic (reductive) conditions. For the compounds to act as HAP’s and exert a therapeutic effect, they should exhibit one-electron reduction potentials (*E*^1^) appropriate for efficient activation by reductases in hypoxic regions, but also be susceptible to reoxidation by molecular oxygen to prevent undesirable toxicity in normal cells. The rate-limiting step for the initial reduction of nitroaromatics is believed to be the first step: Reduction of the -NO_2_ group to a nitroso compound, -N=O [21]. As a comparative example, the reduction potentials for nitrobenzenes typically fall within the range of −0.58 to −0.81 V vs. NHE, while nitrosobenzenes exhibit reduction potentials in the range of +0.04 to +0.25 V [22]. As a result, we can infer that the experimental one-electron reduction potential of our nitrobenzyl compounds describe the rate of the first reduction step for activation of the SN-38 analogs, and can serve as a surrogate measure for the reductive prodrug-ability of the potential HAP’s. Too low of a reduction potential would hinder the reductive activation of the compounds, particularly if it was significantly lower than those of the reductase enzymes that typically mediate this process (*E*^1^ = −220 to −310 mV). Contrastingly, too high a reduction potential would render the reduction too fast and impact the reversibility of the reaction under normal oxygen conditions, potentially negating any hypoxia-selective behavior of the prodrug. The effective *E*^1^ of oxygen (O_2_) is −155 mV vs. NHE [23] and a prodrug with a reduction potential higher than this (closer to zero) would not serve as an effective HAP. According to the literature, a multiple linear regression analysis of 35 nitroaromatics dictates that an increase of 100 mV in reduction potential can increase potency 10-fold, but also aerobic toxicity as well [24]. For compounds to serve as effective HAP, an empirical range of reductions potentials of −400 to −200 mV has been established [13,21], in addition to a narrower window of −450 to −300 mV [25,26]. Additionally, to avoid the potential of observing toxic effects in normal tissues which can be mildly hypoxic under physiological conditions (such as bone marrow [27,28,29], esophagus [30], retina [31] and skin [32]), higher *E*^1^ should be avoided. In order to achieve effective targeting of severe hypoxia which can be specific to tumor environments, it would be ideal for the first reduction potential to fall within a lower range of −500 to −250 mV.

The reported values for the *E*_1/2_^Fc^ of nitrobenzene and 4-nitrotoluene are −1492 mV and −1547 mV [33], respectively, which are within the range of the values calculated here, −1434 to −1461 mV, for the nitrobenzyl derivatives. Care should be taken, however, when comparing different methods, such as cyclic voltammetry and pulse radiolysis, and the *E*_1/2_^NHE^ and *E*^1^ values. For instance, the *E*_1/2_^NHE^ for nitrobenzene is −792 mV when converted from *E*_1/2_^Fc^ using Equation (3), which is different than the *E*^1^ value of −486 mV vs. NHE measured using pulse radiolysis [34]. However, these values can be utilized and compared when considering the relative reduction potentials between differing nitroaromatics, i.e., the relative ease of reduction. In comparison, the one-electron reduction potentials *E*^1^ of the clinically advanced HAP TH-302 is −407 mV vs. NHE (pulse radiolysis) [35], while the half-wave reduction potentials *E*_1/2_^Ag^ for the HAP KS119 have been reported to be −415 and −575 mV vs. Ag/Ag^+^, as measured on a racemic mixture by differential pulse polarography [36]. The half-wave reduction potentials of the three nitrobenzyl-SN-38 analogs were found to be much lower than those of TH-302 and KS119 (*E*_1/2_^NHE^ = −761 to −734 mV, *E*_1/2_^Ag^ = −991 to −969 mV). These values for the SN-38 analogs (as highlighted earlier for *E*_1/2_^Fc^) are more similar to those reduction potentials that would be expected for 2-nitrobenzene and 2-nitrotoluene.

Biologically, HAP’s are reduced by one-electron reductases, and the redox properties of these enzymes are an important consideration when designing suitable HAP’s that would be expected to target hypoxic tumor environments. Previously, four reductases have been studied for their ability to mediate the prodrug ability of hypoxia targeting agents, such as NADPH:cytochrome P450 oxidoreductase (POR) [37], methionine synthase reductase (MTRR) [38], NADPH-dependent diflavin oxidoreductase 1 (NDOR) [39] and inducible nitric oxide synthase (iNOS) [40]. The substrates for these enzymes can receive an electron from the flavin mononucleotide (FMN) catalytic site [41], and the reduction potentials have been found to be −305, −269, −245 and −227 mV, respectively, for NDOR1 [42], POR [43], iNOS [44] and MTRR [45] (vs. NHE, measured by potentiometric titration). In comparison to the *E*_1/2_^NHE^ values (−761 to −734 mV) of the nitrobenzyl-SN-38 derivatives (Table 1), the enzymatic reduction potential values are considerable higher, and one would question whether the potential HAP analogs of SN-38 designed herein would be effectively reduced enzymatically. At physiological conditions, however, the reductases utilize NAD(P)H as the electron donor [41] and can mediate this reductive process, even when the reductions potential for an HAP is not within range of the enzymatic threshold. For instance, the reduction potential of TH-302 was found to be −407 mV, which is lower than that of the reductase enzymes, however, this compound has been shown to be reduced in hypoxic environments. What can be deduced however, is that the reduction potentials for the nitrobenzyl-SN-38 derivatives are well below that of the reductase enzymes and other clinically advanced HAP’s (such as TH-302), and it would be expected that the novel HAP’s designed here would undergo a slower rate of reductive metabolism in hypoxic environments. As a result, the cyclic voltammetry experiments indicate that a need exists to increase the values of the reduction potentials in order to bring the values within range of the enzymatic processes (−450 to −300 mV) in order to develop clinically viable HAP of SN-38.

Furthermore, the reversibility of the initial one-electron reduction is a critical component in the ability of these prodrugs to target hypoxia in tumors. Under normoxic conditions, the inter-transition between the nitro prodrug and its radical anion is a key element of the formation of the futile cycle that is required for the HAP-activating process. In other words, the reversibility of the first reductive reaction (the reaction of the radical anion product back to the nitro compound mediated by molecular oxygen) is required for the prodrug to remain intact under normal oxygen conditions, yet serves as a means to target hypoxia in the absence of oxygen and lead to the reductive fragmentation of the trigger and effector. The peak current ratio (*i*_pa_/*i*_pc_; Table 1) can provide some insight into the extent of reversibility. The closer the ratio is to 1, the more reversible the reaction is under experimental conditions. The peak current ratios were found to be 0.589, 0.548 and 0.348 for the 2-, 3- and 4-nitrobenzyl-SN-38 analogs, respectively. This indicates that the reactions are quasi-reversible and validates the potential of the novel HAP’s to participate in the futile redox cycle that would enable these compounds to target hypoxic environments, and serves as a proof-of-principle for hypoxia-targeting. Interestingly, there appears to be an inverse relationship between the *i*_pa_/*i*_pc_ ratios and the distance of the nitro substituent from the benzylic carbon (2-nitro closest, 4-nitro farthest). External factors such as solvent, rate of scan, water levels and oxygen levels can influence *i*_pa_/*i*_pc_ values, and the most ‘exposed’ nitro group (4-nitro) may be more subject to these external factors, and influence reversibility.

### 3.3. Reduction of Prodrug

**Figure 5:**
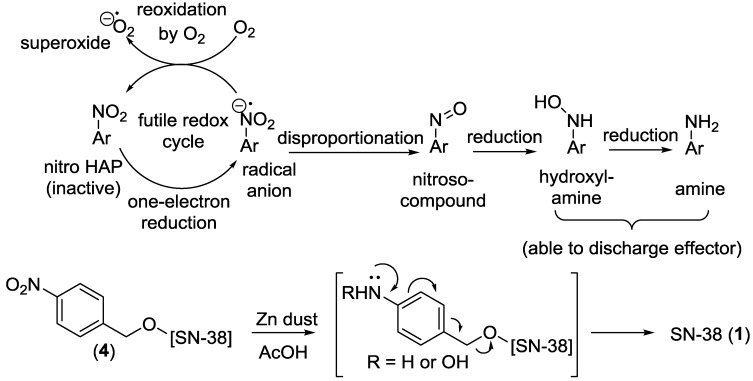
Simplified nitro-HAP-activating process for prodrug **4** (Ar = substituted aromatic ring).

In order to serve as prodrugs, the nitrobenzyl ‘triggers’ must be reduced under physiological conditions and release the ‘effector’ SN-38. Under normoxic conditions, a one-electron reduction of the nitro prodrug leads to the generation of its radical anion, which is then reoxidized via oxygen, leading to the formation of the futile redox cycle. Under hypoxic conditions, the radical anion persists, and further reduction leads to the formation of an amine (or hydroxylamine) that is sufficiently electron-donating, leading to the fragmentation of the trigger and effector, Figure 5. We have been able to establish the proof-of-principle, via chemical reduction, that a nitroaromatic analog of SN-38 (compound **4**) can serve as a prodrug and release the pharmaceutically active metabolite **1** under reductive conditions. Using a chemical-reducing method, Zn dust/AcOH was able to result in the formation/release of SN-38 from a crude reaction mixture of the 4-nitro-analog **4**, as monitored by LC-MS (see **Supplementary Materials**), and was shown to evolve over time as the reaction progressed (increased production of SN-38). It is not surprising that a reduced form of SN-38 was also observed, either through the over-reduction of the starting material, followed by reductive fragmentation of the trigger-effector, or via the direct reduction of SN-38. Overall, the chemical reduction of the nitroaromatic analog **4** and the subsequent release of SN-38 highlights that this compound can serve as a prodrug. Coupled with the cyclic voltammetry results, and the validation of the prodrug ability of compound **4**, these results further establish a proof-of-concept that the nitroaromatic C-10-tethered analogs of SN-38 can serve as HAP’s. In the next stages of our research project, we expect to study this reductive process (prodrug release) under hypoxic conditions with tumor cells, as well to explore the hypoxia-dependent metabolism in a cell-free system, such as a liver S9 fraction. Additionally, we also hope to explore the direct enzymatic reduction of the prodrugs by the nitroreductases listed above under hypoxic conditions, and whether or not these analogs can serve as substrates for hypoxia-independent reductases such as AKR1C3 or NQO1, under normoxic conditions.

## 4. Materials and Methods

### 4.1. General Information

^1^H and ^13^C nuclear magnetic resonance (NMR) spectra were recorded on a Bruker 400 MHz spectrometer, Billerica, MA, USA (400 and 101 MHz, respectively) using DMSO-*d*_6_ (Merck KGaA, Darmstadt, Germany) as solvent with tetramethylsilane (TMS) as an internal standard. Liquid chromatography-mass spectrometry (LC-MS) analyses were performed on a Shimadzu LC-MS spectrometer, Kyoto, Japan. SN-38 (7-ethyl-10-hydroxylcamptothecin, purity 95%+) was purchased from Ark Pharm, Inc., Arlington Heights, IL, USA. 1,8-Diazabicyclo[5.4.0]undec-7-ene (DBU); 2-, 3- and 4-nitrobenzyl bromides were purchased from Sigma-Aldrich, St. Louis, MO, USA. Organic solvents were ordered from BDH, VWR Analytical unless specified otherwise. All chemicals were used without further purification unless otherwise indicated.

### 4.2. Synthesis and Characterization of 2-Nitrobenzyl-C_10_-SN-38 *(**2**)*

SN-38 (**1**) (0.0941 g, 0.24 mmol) was added into a round bottom flask (10 mL) with a magnetic stirring bar. Dimethylformamide (DMF, 2.0 mL, anhydrous, Sigma-Aldrich) and DBU (80 µL, 0.54 mmol) were then added, and the mixture was sonicated until SN-38 was dissolved. The dissolved SN-38 was then stirred on a stirring plate and purged with argon flow for 20 min. 2-Nitrobenzyl bromide (0.1149 g, 0.53 mmol) was dissolved in 0.75 mL anhydrous DMF and then added slowly (over 30 min) into SN-38 with a syringe. The mixture was allowed to stir at room temperature (22 °C) for 6 h. The resulted yellow solid was separated by centrifuging and washed with acetone (2 mL × 5). Residual acetone was then evaporated under vacuum to give 2-nitrobenzyl SN-38 as a yellow powder (0.0986 g, 78%). ^1^H-NMR (400 MHz, DMSO) δ 8.21–8.06 (m, 2H), 7.89 (d, *J* = 7.5 Hz, 1H), 7.81 (t, *J* = 7.3 Hz, 1H), 7.66 (t, *J* = 7.4 Hz, 1H), 7.59 (d, *J* = 7.7 Hz, 2H), 7.28 (s, 1H), 6.52 (s, 1H), 5.72 (s, 2H), 5.43 (s, 2H), 5.31 (s, 2H), 3.17 (q, *J* = 7.4 Hz, 2H), 1.87 (m, *J* = 14.2, 6.9 Hz, 2H), 1.23 (t, *J* =7.5 Hz, 3H), 0.88 (t, *J* = 7.3 Hz, 3H). ^13^C-NMR (101 MHz, DMSO) δ 173.00, 157.31, 157.14, 150.53, 150.44, 148.31, 146.70, 145.10, 144.48, 134.41, 132.14, 130.25, 129.92, 129.00, 128.19, 125.34, 122.73, 118.84, 104.64, 99.98, 96.56, 72.86, 67.32, 65.71, 50.02, 30.68, 22.67, 13.86, 8.23. ESI-MS(+) *m*/*z* (% relative intensity, [ion]): 528.20 (100, [M + H]^+^), 569.25 (57.55, [M + H + CH_3_CN]^+^).

### 4.3. Synthesis and Characterization of 3-Nitrobenzyl-C_10_-SN-38 *(**3**)*

The synthesis was conducted in a microwave synthesizer (Discover^®^SP W/Activent, CEM, Matthews, NC, USA). To a microwave reaction vessel (10 mL) were added SN-38 (**1**) (0.0241 g, 0.061 mmol), dry dichloromethane (DCM, 4 mL, dried over molecular sieves overnight), DBU (36 µL, 0.24 mmol) and 3-nitrobenzyl bromide (0.0375 g, 0.17 mmol). The reaction vessel was then sealed and placed in the microwave synthesizer. The reaction was conducted under dynamic mode, 66 °C, PowerMax mode (simultaneous air cooling, model of microwave synthesizer: Discover^®^SP W/Activent, CEM, USA), max power 200 W, max pressure 300 psi, for 10 min. The reaction mixture was then purified with silica gel column chromatography on a CombiFlash^®^ Rf 200 purification system, Teledyne Isco, Lincoln, NE, USA, with ethyl acetate in hexane from 0% to 100% (3-nitrobenzyl SN-38 was eluted out with 100% ethyl acetate). Residual solvent was evaporated under vacuum to give 3-nitrobenzyl SN-38 as a yellow powder (0.0216 g, 67%). ^1^H-NMR (400 MHz, DMSO) δ 8.46 (s, 1H), 8.24 (dd, *J* = 8.2, 1.5 Hz, 1H), 8.17–8.08 (m, 1H), 8.04 (d, *J* = 7.8 Hz, 1H), 7.75 (t, *J* = 7.9 Hz, 1H), 7.64 (dd, *J* = 6.7, 3.1 Hz, 2H), 7.28 (s, 1H), 6.53 (s, 1H), 5.55 (s, 2H), 5.43 (s, 2H), 5.31 (s, 2H), 3.20 (q, *J* = 7.3 Hz, 2H), 1.96–1.77 (m, 2H), 1.24 (t, *J* = 7.6 Hz, 3H), 0.88 (t, *J* = 7.3 Hz, 3H). ^13^C-NMR (101 MHz, DMSO) δ 173.00, 157.27, 157.19, 150.50, 150.27, 148.33, 146.67, 145.00, 144.40, 139.54, 134.75, 132.05, 130.66, 128.89, 128.15, 123.37, 122.96, 122.79, 118.78, 104.35, 96.54, 72.86, 68.88, 65.71, 49.97, 30.69, 22.67, 13.85, 8.23. ESI-MS(+) *m*/*z* (% relative intensity, [ion]): 528.20 (100, [M + H]^+^), 569.30 (69.21, [M + H + CH_3_CN]^+^).

### 4.4. Synthesis and Characterization of 4-Nitrobenzyl-C_10_-SN-38 *(**4**)*

SN-38 (**1**) (0.0936 g, 0.24 mmol) was added into a round bottom flask (10 mL) with a magnetic stirring bar. Dimethylformamide (DMF, 2.0 mL, anhydrous, Sigma-Aldrich) and DBU (80 µL, 0.54 mmol) were then added, and the mixture was sonicated until SN-38 was dissolved. The dissolved SN-38 was then stirred on a stirring plate and purged with argon flow for 20 min. 4-Nitrobenzyl bromide (0.1187 g, 0.55 mmol) was dissolved in 0.75 mL anhydrous DMF and then added slowly (over 30 min) into SN-38 with a syringe. The mixture was allowed to stir at room temperature (22 °C) for 6 h. The resulted yellow solid was separated by centrifuging and washed with acetone (2 mL × 5). Residual acetone was then evaporated under vacuum to give 4-nitrobenzyl SN-38 as a yellow powder (0.0864 g, 68%). ^1^H-NMR (400 MHz, DMSO) δ 8.35–8.26 (m, 2H), 8.13 (d, *J* = 9.1 Hz, 1H), 7.85 (d, *J* = 8.8 Hz, 2H), 7.63 (dt, *J* = 5.7, 2.6 Hz, 2H), 7.28 (s, 1H), 6.53 (s, 1H), 5.56 (s, 2H), 5.43 (s, 2H), 5.31 (s, 2H), 3.18 (q, *J* = 7.4 Hz, 2H), 1.87 (m, *J* = 14.0, 7.1 Hz, 2H), 1.25 (t, *J* = 7.6 Hz, 3H), 0.88 (t, *J* = 7.3 Hz, 3H). ^13^C-NMR (101 MHz, DMSO) δ 172.98, 157.33, 157.25, 150.54, 150.39, 147.61, 146.72, 145.10, 145.08, 144.49, 132.12, 128.98, 128.23, 124.16, 122.94, 118.84, 104.55, 96.56, 72.87, 71.45, 69.07, 65.74, 50.00, 30.76, 22.68, 13.89, 8.21. ESI-MS(+) *m*/*z* (% relative intensity, [ion]): 528.25 (100, [M + H]^+^), 569.30 (92.39, [M + H + CH_3_CN]^+^).

### 4.5. Topoisomerase I Inhibitory Assay

The method used has been previously validated by the Hasinoff group [46,47]. The recombinant human topoisomerase 1, pBR322 DNA, and assay buffer were from TopoGEN, Inc., Buena Vista, CO, USA. Each 20 μL of assay mixture contained 50 ng of pBR322 DNA, 0.5 units of topoisomerase 1 (except the mixture for the control lane “no top1”) and tested drugs of indicated final concentrations. The order of addition was assay buffer, DNA, drug, and then topoisomerase 1. After incubation at 37 ⁰C in assay buffer for 30 min, the reaction was terminated with 0.5% (*v*/*v*) SDS and 25 mM Na_2_EDTA. Electrophoresis was carried out at 8 V/cm for 1 h on a plate of agarose gel (1.2%, *w*/*v*). The gel plate was then stained with ethidium bromide for 20 min and immersed in water for 24 h to elute the drugs (SN-38 and its derivatives emit strong fluorescence under UV light, impairing the quality of image). The gel plate was then visualized by UV light, and the emitted fluorescence was recorded on an Alpha Innotech (San Leandro, CA, USA) Fluorochem 8900 imaging system equipped with a 365-nm UV illuminator and a charge-coupled device camera.

### 4.6. Cell Viability Assay

Human leukemia K562 cells were obtained from the American Type Culture Collection and maintained as suspension cultures in Dulbecco’s modified Eagle’s medium (Invitrogen, Carlsbad, CA, USA) containing 4 mM l-glutamine and supplemented with 20 mM HEPES (Sigma-Aldrich, St. Louis, MO, USA), 10% fetal calf serum (Invitrogen), 100 units/mL penicillin G, and 100 μg/mL streptomycin in an atmosphere of 5% CO_2_ and 95% air at 37 °C, pH 7.4. Cells in exponential growth were harvested and seeded with 1150 cells/well in 96-well microtiter plates (100 μL/well). Tested drugs were dissolved in dimethyl sulfoxide with 11 different concentrations to construct the dose-response curves and were added to give a final volume of 200 μL/well. Each drug was tested in two adjacent lanes of wells. The final concentration of dimethyl sulfoxide did not exceed 0.5% (*v*/*v*), and it was an amount that had no detectable effect on cell growth. The cells were incubated with tested drugs for 48 h and then assayed with MTS on a spectrophotometer (SpectraMax 190, Molecular Devices, San Jose, CA, USA). Values of half maximal inhibitory concentrations (IC_50_) were obtained by fitting the average absorbance-concentration data of two lanes to a four-parameter logistic equation (SigmaPlot, Jandel, San Rafael, CA, USA).

### 4.7. Reduction of Prodrug 4-Nitrobenzyl-C_10_-SN-38 *(**4**)*

Approximately 15 mL of ethanol and 5 mL of distilled water were purged of oxygen by bubbling argon through the liquids for 20 min in vented septum sealed vials. 4-Nitrobenzyl-C_10_-SN-38 (**4**) (11.1 mg, 0.021 mmol) was dissolved in 6 mL of deoxygenated ethanol, followed by the addition of 2 mL of deoxygenated water, in a 20 mL vented septum sealed vial, purged with argon. Zinc dust (11.0 mg, 0.168 mmol) was then added, followed by reagent grade acetic acid (5 µL, 0.084 mmol). The reaction was allowed to stir under argon at room temperature. After 10 min, an aliquot of the crude reaction mixture was filtered and re-suspended in a 1:1 mixture of DMSO:acetonitrile and subjected to LC-MS analysis. Following 8 hours, an additional aliquot of the crude reaction mixture was filtered and re-suspended in a 1:1 mixture of DMSO:acetonitrile and subjected to LC-MS analysis. Chromatography was performed using a Hypersil GOLD 150 × 4.6 mm (5 µm) column running a gradient of 0.1% formic acid in acetonitrile [0 min 5%, 1 min 5%, 15 min 95%, 19 min 95%, 20 min 5%, 23 min 5%], with the MS set in the ESI positive mode. The LC-MS chromatogram at 10 min indicated the presence of both SN-38 and a reduced form of SN-38, as well as unreacted starting material, as indicated by the representative mass spectrums of the subsequent LC peaks. After 8 h of reaction, a LC-MS chromatogram of the crude reaction mixture indicated an increased concentration of SN-38, in addition to unreacted starting material, as well as some reduced SN-38.

## 5. Conclusions

The C-10 substituted 2-, 3- and 4-nitrobenzyl derivatives of SN-38 have been successfully synthesized in good yields, 78%, 67% and 68%, respectively. The synthetic procedure involved using either a room temperature or microwave-mediated alkylation reaction from the corresponding nitrobenzyl bromides, under basic conditions. A cell viability assay using human leukemia K562 cells indicated that the analogs of SN-38 possessed anticancer properties with measured IC_50_’s between 12 and 58 nM. These values represented an 8-, 4- and 19-fold decrease in cytotoxicity for the 2-, 3- and 4-nitrobenzyl analogs when compared to SN-38 (3.0 nM IC_50_), which meets the principle of prodrug design. A topoisomerase I inhibition assay indicated that at a 20 µM concentration, the 4-nitrobenzyl analog inhibited the ability of topoisomerase I to relax pBR322 supercoiled DNA, while the 2-nitrobenzyl analog did show topoisomerase I inhibition at 20 µM. The results from the cyclic voltammetry experiments indicate that the reduction potentials of the three nitrobenzyl analogs of SN-38 were lower than those reported for other clinically explored HAP’s, and were on par with those reported for simple nitroaromatics. The results also suggest that the reduction of the three analogs was partly reversible under the testing conditions, and indicates the potential for these molecules to serve as HAP, provided that they are substrates of cellular reductases, via the formation of a futile redox cycle with oxygen under normoxic conditions. The prodrug ability of the 4-nitro analog (**4**) was validated by chemical reduction using Zn dust and acetic acid, as evidenced by the evolution of SN-38, and further serves to infer the ability of this class of compounds to serve as HAP’s.

Further examination of these SN-38 analogs under hypoxic conditions with tumor cells is warranted to investigate the process of reductive activation and the prospect of clinical application. The results also suggest that the next generation of SN-38 HAP’s should possess bulkier nitroaromatic groups to reduce the cytotoxicity even further, and contain triggers that possess higher reduction potentials in order to bring the compounds within range of cellular reductases (−450 to −300 mV). We have established, however, the proof-of-principle that we can develop potential hypoxia-activated prodrugs of SN-38 via the attachment of triggers (nitroaromatics) to the C-10 position of SN-38. These analogs have been shown to possess reduced cytotoxicity, and inhibit topoisomerase I at a comparable concentration to SN-38, which meets a basic principle of prodrug design. The reduction potentials were found to be lower than other literature HAP’s, although partly reversible, and validates the ability of these prodrugs to potentially serve as hypoxia-targeting therapeutics.

## Figures and Tables

**Table 1:** Results of cyclic voltammetry of SN-38 analogs.

	***E*_pa_^Ag^/mV**	***i*_pa_/µA**	***E*_pc_^Ag^/mV**	***i*_pc_/µA**	***E*_pa_^Ag^(Fc)/mV**	***E*_pc_^Ag^(Fc)/mV**
2-Nitrobenzyl- (**2**)	−948	5.81	−1033	−9.86	427	514
3-Nitrobenzyl- (**3**)	−929	5.55	−1043	−10.14	405	509
4-Nitrobenzyl- (**4**)	−882	4.17	−1055	−11.97	427	504
**Cont’d**	***i*_pa_/*i*_pc_**	***E*_1/2_^Ag^(Fc)/mV**	***E*_1/2_^Ag^/mV**	***E*_1/2_^Fc^/mV**	***E*_1/2_^NHE^/mV**	
(**2**)	0.589	471	−991	−1461	−761	
(**3**)	0.548	457	−986	−1443	−743	
(**4**)	0.348	466	−969	−1434	−734	

## Supplementary Materials

Supplementary materials with additional experimental details are available on line.

## Abbreviations

The following abbreviations are used in this manuscript:

DBU	Diazabicyclo[5.4.0]undec-7-ene
DCM	Dichloromethane
DMSO	Dimethyl Sulfoxide
FMN	Flavin Mononucleotide
iNOS	Inducible Nitric Oxide Synthase
MDPI	Multidisciplinary Digital Publishing Institute
MTRR	Methionine Synthase Reductase
NDOR	NADPH Dependent Diflavin Oxidoreductase 1
NHE	Normal Hydrogen Electrode
POR	NADPH:cytochrome P450 Oxidoreductase
SCE	Saturated Calomel Electrode
^1^H-NMR	Proton Nuclear Magnetic Resonance
^13^C-NMR	Carbon Nuclear Magnetic Resonance

## References

[1] Pizzolato J.F., Saltz L.B. (2003). The camptothecins. Lancet.

[2] Höckel M., Vaupel P. (2001). Tumor hypoxia: Definitions and current clinical, biologic, and molecular aspects. J. Natl. Cancer Inst..

[3] Vaupel P., Kallinowski F., Okunieff P. (1989). Blood Flow, Oxygen and Nutrient Supply, and Metabolic Microenvironment of Human Tumors: A Review. Cancer Res..

[4] Petrova V., Annicchiarico-Petruzzelli M., Melino G., Amelio I. (2018). The hypoxic tumour microenvironment. Oncogenesis.

[5] Semenza G.L. (2016). The hypoxic tumor microenvironment: A driving force for breast cancer progression. Biochim. Biophys. Acta (BBA) Mol. Cell Res..

[6] Höckel M., Schlenger K., Aral B., Mitze M., Schäffer U., Vaupel P. (1996). Association between tumor hypoxia and malignant progression in advanced cancer of the uterine cervix. Cancer Res..

[7] Wilson W.R., Hay M.P. (2011). Targeting hypoxia in cancer therapy. Nat. Rev. Cancer.

[8] Cater D.B., Phillips A.F. (1954). Measurement of electrode potentials in living and dead tissues. Nature.

[9] Jiang J., Auchinvole C., Fisher K., Campbell C.J. (2014). Quantitative measurement of redox potential in hypoxic cells using SERS nanosensors. Nanoscale.

[10] Mistry I.N., Thomas M., Calder E.D.D., Conway S.J., Hammond E.M. (2017). Clinical Advances of Hypoxia-Activated Prodrugs in Combination With Radiation Therapy. Int. J. Radiat. Oncol. Boil. Phys..

[11] Denny W.A., Wilson W.R., Hay M.P. (1996). Recent developments in the design of bioreductive drugs. Br. J. Cancer Suppl..

[12] Sum B.G., Denny W.A., Wilson W.R. (1997). Nitro reduction as an electronic switch for bioreductive drug activation. Oncol. Res..

[13] Wardman P. (2001). Electron transfer and oxidative stress as key factors in the design of drugs selectively active in hypoxia. Curr. Med. Chem..

[14] Liang D., Miller G.H., Tranmer G.K. (2015). Hypoxia activated prodrugs: Factors influencing design and development. Curr. Med. Chem..

[15] Pouysségur J., Dayan F., Mazure N.M. (2006). Hypoxia signalling in cancer and approaches to enforce tumour regression. Nature.

[16] Connelly N.G., Geiger W.E. (1996). Chemical redox agents for organometallic chemistry. Chem. Rev..

[17] Pavlishchuk V.V., Addison A.W. (2000). Conversion constants for redox potentials measured versus different reference electrodes in acetonitrile solutions at 25 °C. Inorg. Chim. Acta.

[18] Sheng G., Wu X., Cai X., Zhang W. (2015). Cooperation of a Reductant and an Oxidant in One Pot To Synthesize Amides from Nitroarenes and Aldehydes. Synthesis.

[19] Porzelle A., Woodrow M.D., Tomkinson N.C.O. (2009). Facile Procedure for the Synthesis of *N*-Aryl-*N*-hydroxy Carbamates. Synlett.

[20] Kelly S.M., Lipshutz B.H. (2014). Chemoselective Reductions of Nitroaromatics in Water at Room Temperature. Org. Lett..

[21] Denny W.A., Wilson W.R. (1986). Considerations for the Design of Nitrophenyl Mustards as Agents with Selective Toxicity for Hypoxic Tumor Cells. J. Med. Chem..

[22] Kovacic P., Kassel M.A., Feinberg B.A., Corbett M.D., McClelland R.A. (1990). Reduction potentials in relation to physiological activities of benzenoid and heterocyclic nitroso compounds: Comparison with the nitro precursors. Bioorg. Chem..

[23] Wardman P. (1985). Some reactions and properties of nitro radical-anions important in biology and medicine. Environ. Health Perspect..

[24] Beveridge A.J., Williams M., Jenkins T.C. (1996). Calculation of one-electron reduction potentials for nitroheterocyclic hypoxia-selective agents. J. Chem. Soc.-Faraday Trans..

[25] Mason R.P., Holtzman J.L. (1975). The role of catalytic superoxide formation in the O_2_ inhibition of nitroreductase. Biochem. Biophys. Res. Commun..

[26] Denny W.A., Wilson W.R. (1993). Bioreducible mustards: a paradigm for hypoxia-selective prodrugs of diffusible cytotoxins (HPDCs). Cancer Metastasis Rev..

[27] Allalunis M.J., Chapman J.D., Turner A.R. (1983). Identification of a hypoxic population of bone marrow cells. Int. J. Radiat. Oncol. Boil. Phys..

[28] Nombela-Arrieta C., Pivarnik G., Winkel B., Canty K.J., Harley B., Mahoney J.E., Park S.Y., Lu J., Protopopov A., Silberstein L.E. (2013). Quantitative imaging of haematopoietic stem and progenitor cell localization and hypoxic status in the bone marrow microenvironment. Nat. Cell Boil..

[29] Parmar K., Mauch P., Vergilio J.A., Sackstein R., Down J.D. (2007). Distribution of hematopoietic stem cells in the bone marrow according to regional hypoxia. Proc. Natl. Acad. Sci. USA.

[30] Parliament M.B., Franko A.J., Wiebe L.I. (1992). Nitroimidazole adducts as markers for tissue hypoxia: Mechanistic studies in aerobic normal tissues and tumour cells. Br. J. Cancer.

[31] Lee A.E., Wilson W.R. (2000). Hypoxia-dependent retinal toxicity of bioreductive anticancer prodrugs in mice. Toxicol. Appl. Pharmacol..

[32] Evans S.M., Schrlau A.E., Chalian A.A., Zhang P., Koch C.J. (2006). Oxygen levels in normal and previously irradiated human skin as assessed by EF5 binding. J. Investig. Dermatol..

[33] Kuhn A., Von Eschwege K.G., Conradie J. (2012). Reduction potentials of para-substituted nitrobenzenes-an infrared, nuclear magnetic resonance, and density functional theory study. J. Phys. Org. Chem..

[34] Wardman P. (1989). Reduction Potentials of One Electron Couples Involving Free Radicals in Aqueous Solution. J. Phys. Chem. Ref. Data.

[35] Meng F., Evans J.W., Bhupathi D., Banica M., Lan L., Lorente G., Duan J.X., Cai X., Mowday A.M., Guise C.P. (2012). Molecular and cellular pharmacology of the hypoxia-activated prodrug TH-302. Mol. Cancer Ther..

[36] Penketh P.G., Baumann R.P., Shyam K., Williamson H.S., Ishiguro K., Zhu R., Eriksson E.S.E., Eriksson L.A., Sartorelli A.C. (2011). 1,2-Bis(methylsulfonyl)-1-(2-chloroethyl)-2-[[1-(4-nitrophenyl)ethoxy]carbonyl]hydrazine (KS119): A Cytotoxic Prodrug with Two Stable Conformations Differing in Biological and Physical Properties. Chem. Boil. Drug Des..

[37] Su J., Gu Y., Pruijn F.B., Smaill J.B., Patterson A.V., Guise C.P., Wilson W.R. (2013). Zinc finger nuclease knock-out of NADPH:cytochrome P450 oxidoreductase (POR) in human tumor cell lines demonstrates that hypoxia-activated prodrugs differ in POR dependence?. J. Boil. Chem..

[38] Guise C.P., Abbattista M.R., Tipparaju S.R., Lambie N.K., Su J., Li D., Wilson W.R., Dachs G.U., Patterson A.V. (2012). Diflavin oxidoreductases activate the bioreductive prodrug PR-104A under hypoxia. Mol. Pharmacol..

[39] Paine M.J.I., Garner A.P., Powell D., Sibbald J., Sales M., Pratt N., Smith T., Tew D.G., Wolf C.R. (2000). Cloning and characterization of a novel human dual flavin reductase. J. Boil. Chem..

[40] Garner A.P., Paine M.J.I., Rodriguez-Crespo I., Chinje E.C., De Montellano P.O., Stratford I.J., Tew D.G., Wolf C.R. (1999). Nitric oxide synthases catalyze the activation of redox cycling and bioreductive anticancer agents. Cancer Res..

[41] Aigrain L., Fatemi F., Frances O., Lescop E., Truan G. (2012). Dynamic control of electron transfers in diflavin reductases. Int. J. Mol. Sci..

[42] Finn Robert D., Basran J., Roitel O., Wolf C.R., Munro Andrew W., Paine Mark J.I., Scrutton Nigel S. (2003). Determination of the redox potentials and electron transfer properties of the FAD- and FMN-binding domains of the human oxidoreductase NR1. Eur. J. Biochem..

[43] Munro A.W., Noble M.A., Robledo L., Daff S.N., Chapman S.K. (2001). Determination of the redox properties of human NADPH-cytochrome P450 reductase. Biochemistry.

[44] Gao Y.T., Smith S.M.E., Weinberg J.B., Montgomery H.J., Newman E., Guillemette J.G., Ghosh D.K., Roman L.J., Martasek P., Salerno J.C. (2004). Thermodynamics of Oxidation-Reduction Reactions in Mammalian Nitric-oxide Synthase Isoforms. J. Boil. Chem..

[45] Wolthers K.R., Basran J., Munro A.W., Scrutton N.S. (2003). Molecular dissection of human methionine synthase reductase: Determination of the flavin redox potentials in full-length enzyme and isolated flavin-binding domains. Biochemistry.

[46] Liang H., Wu X., Yalowich J.C., Hasinoff B.B. (2008). A three-dimensional quantitative structure-activity analysis of a new class of bisphenol topoisomerase IIα inhibitors. Mol. Pharmacol..

[47] Hasinoff B.B., Creighton A.M., Kozlowska H., Thampatty P., Allan W.P., Yalowich J.C. (1997). Mitindomide is a catalytic inhibitor of DNA topoisomerase II that acts at the bisdioxopiperazine binding site. Mol. Pharmacol..

