# Supplementary material for: Evaluation of Nitrobenzyl Derivatives of Camptothecin as Anti-Cancer Agents and Potential Hypoxia Targeting Prodrugs
Source: Molecules. 2018 Aug 15;23(8):2041. doi: 10.3390/molecules23082041 (PMC6222813; doi:10.3390/molecules23082041)

Supporting Information

Evaluation of Nitrobenzyl Derivatives of Camptothecin as Anti-Cancer Agents and Potential Hypoxia Targeting Prodrugs

Dinghua Liang[1], Xing Wu[1], Brian B. Hasinoff[1], David E. Herbert[2] and Geoffrey K. Tranmer[1,2,*]

[1]College of Pharmacy, University of Manitoba, Winnipeg, MB, Canada

[2]Department of Chemistry, University of Manitoba, Winnipeg, MB, Canada

*geoffrey.tranmer@umanitoba.ca

Table of Contents

1. General Information. . . . . .Page 2
2. Characterization of SN-38 (**1**) . . . . .Page 2
3. Synthesis and Characterization of 2-Nitrobenzyl-SN-38 (**2**) . . . . .Page 3-5
4. Synthesis and Characterization of 3-Nitrobenzyl-SN-38 (**3**) . . . . .Page 5-7
5. Synthesis and Characterization of 4-Nitrobenzyl-SN-38 (**4**). . . . . .Page 7-9
6. LC-MS Chromatograms for reduction of 4-Nitrobenzyl-SN-38 (**4**). . . . . .Page 10-13

1. General Information

^{1}H and ^{13}C nuclear magnetic resonance (NMR) spectra were recorded on a Bruker 400 MHz spectrometer (400 and 101 MHz, respectively) using DMSO-d_6 (Merck KGaA, Germany) as solvent with tetramethylsilane (TMS) as an internal standard. Liquid chromatography-mass spectrometry (LC-MS) analyses were performed on a Shimadzu LC-MS spectrometer. SN-38 (7-ethyl-10-hydroxylcamptothecin, purity 95%+) was purchased from Ark Pharm, Inc., USA. 1,8-Diazabicyclo[5.4.0]undec-7-ene (DBU); 2-, 3- and 4-nitrobenzyl bromides were purchased from Sigma-Aldrich. Organic solvents were ordered from BDH, VWR Analytical unless specified otherwise. All chemicals were used without further purification unless otherwise indicated.

2. Characterization of SN-38 (1), (for comparison to synthetic analogs)

^{1}H NMR (400 MHz, DMSO) δ 10.28 (s, 1H), 8.02 (d, *J* = 8.9 Hz, 1H), 7.47 – 7.34 (m, 2H), 7.25 (s, 1H), 6.47 (s, 1H), 5.51 – 5.34 (s, 2H), 5.26 (s, 2H), 3.08 (q, *J* = 7.5 Hz, 2H), 1.94 – 1.75 (m, 2H), 1.30 (t, *J* = 7.6 Hz, 3H), 0.88 (t, *J* = 7.3 Hz, 3H). ^{13}C NMR (101 MHz, DMSO) δ 172.98, 157.32, 157.19, 150.53, 149.32, 146.91, 144.12, 143.21, 132.02, 128.66, 128.45, 122.84, 118.47, 105.25, 96.24, 72.87, 65.73, 49.91, 30.75, 22.74, 13.80, 8.21. ESI-MS(+) *m/z* (% relative intensity, [ion]): 393.20 (100, $[M + H]^+$), 434.25 (58.64, $[M + H + CH_3CN]^+$).

2.1 ^{1}H NMR, ^{13}C NMR, and MS spectra of SN-38 (1)

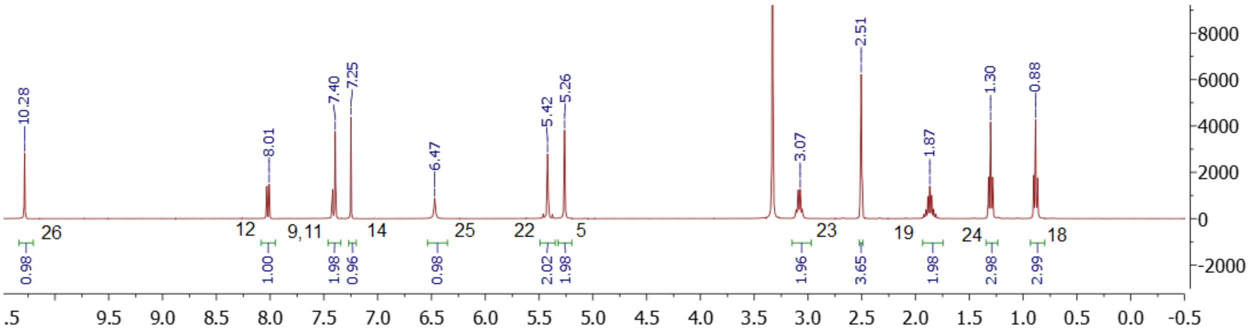

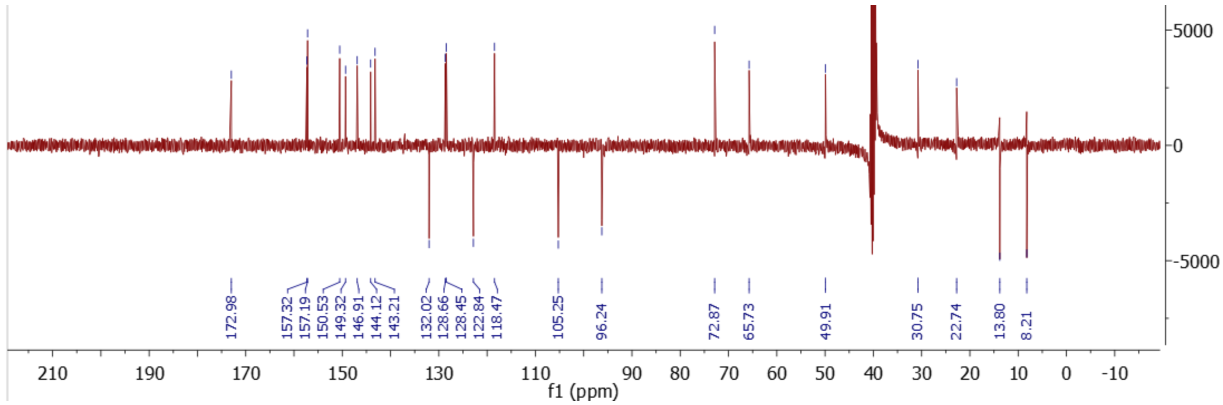

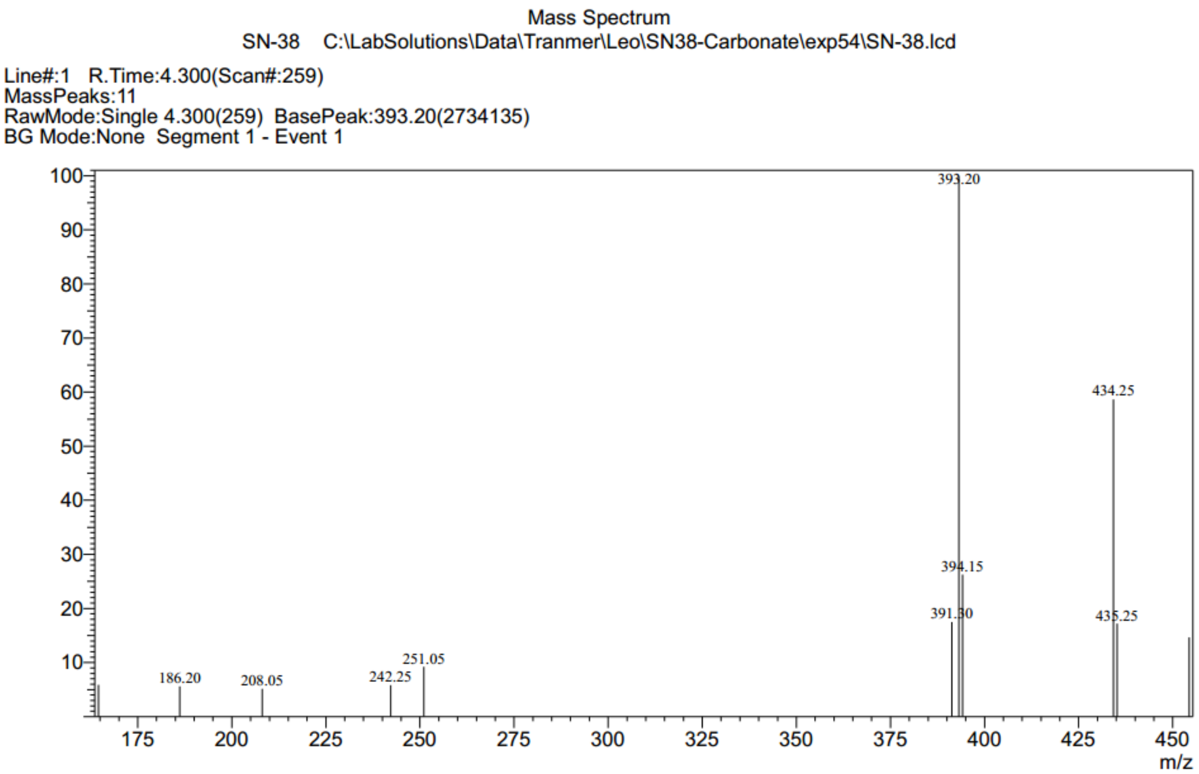

3. Synthesis and Characterization of 2-Nitrobenzyl-SN-38 (2)

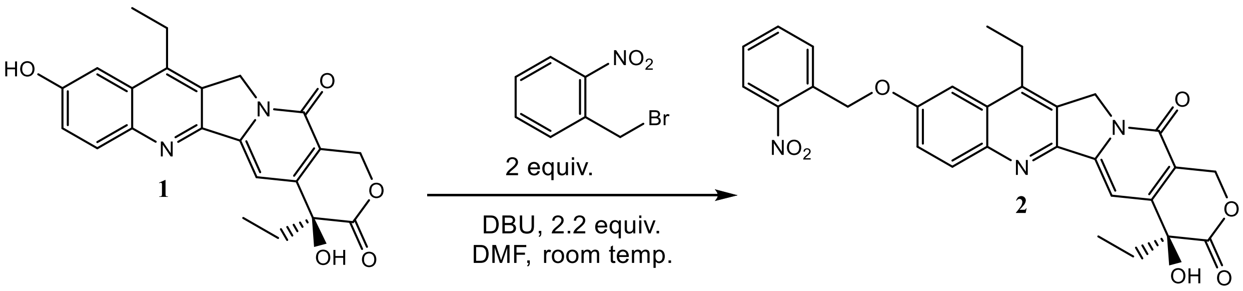

SN-38 (**1**) (0.0941 g, 0.24 mmol) was added into a round bottom flask (10 mL) with a magnetic stirring bar. Dimethylformamide (DMF, 2.0 mL, anhydrous, Sigma-Aldrich) and DBU (80 µL, 0.54 mmol) were then added, and the mixture was sonicated until SN-38 was dissolved. The dissolved SN-38 was then stirred on a stirring plate and purged with argon flow for 20 min. 2-Nitrobenzyl bromide (0.1149 g, 0.53 mmol) was dissolved in 0.75 mL anhydrous DMF and then added slowly (over 30 min) into SN-38 with a syringe. The mixture was allowed to stir at room temperature (22 ^{o}C) for 6 hours. The resulted yellow solid was separated by centrifuging and washed with acetone (2 mL x 5). Residual acetone was then evaporated under vacuum to give 2-nitrobenzyl SN-38 as a yellow powder (0.0986 g, 78%). ^{1}H NMR (400 MHz, DMSO) δ 8.21 – 8.06 (m, 2H), 7.89 (d, *J* = 7.5 Hz, 1H), 7.81 (t, *J* = 7.3 Hz, 1H), 7.66 (t, *J* = 7.4 Hz, 1H), 7.59 (d, *J* = 7.7 Hz, 2H), 7.28 (s, 1H), 6.52 (s, 1H), 5.72 (s, 2H), 5.43 (s, 2H), 5.31 (s, 2H), 3.17 (q, *J* = 7.4 Hz,

2H), 1.87 (m, *J* = 14.2, 6.9 Hz, 2H), 1.23 (t, *J* =7.5 Hz, 3H), 0.88 (t, *J* = 7.3 Hz, 3H). ^{13}C NMR (101 MHz, DMSO) δ 173.00, 157.31, 157.14, 150.53, 150.44, 148.31, 146.70, 145.10, 144.48, 134.41, 132.14, 130.25, 129.92, 129.00, 128.19, 125.34, 122.73, 118.84, 104.64, 99.98, 96.56, 72.86, 67.32, 65.71, 50.02, 30.68, 22.67, 13.86, 8.23. ESI-MS(+) *m/z* (% relative intensity, [ion]): 528.20 (100, $[M + H]^+$), 569,25 (57.55, $[M + H + CH_3CN]^+$).

3.1 ^{1}H NMR, ^{13}C NMR, and MS spectra of 2-nitrobenzyl-SN-38 (2)

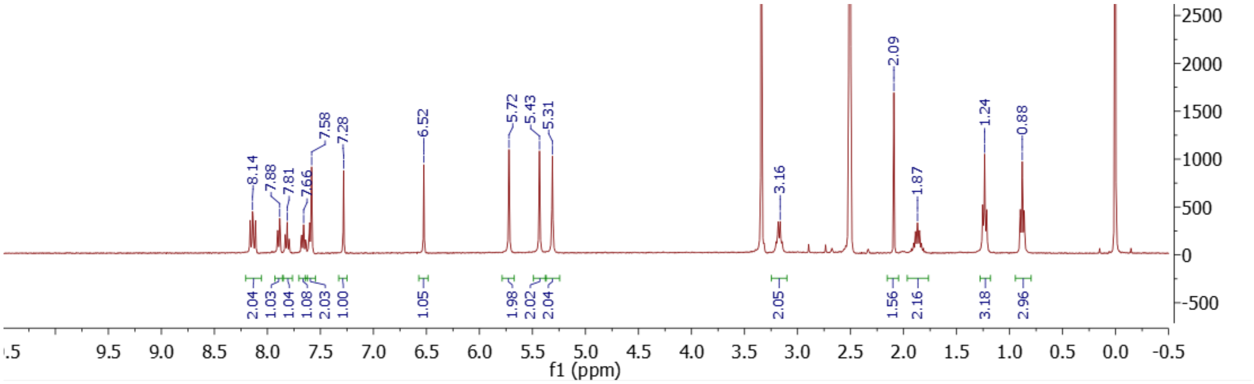

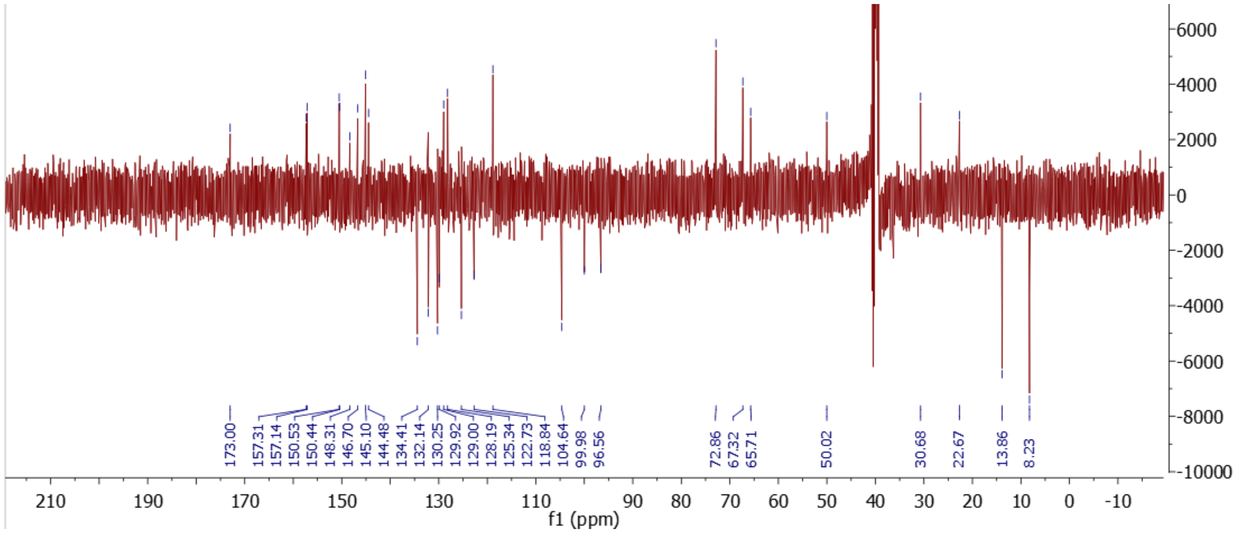

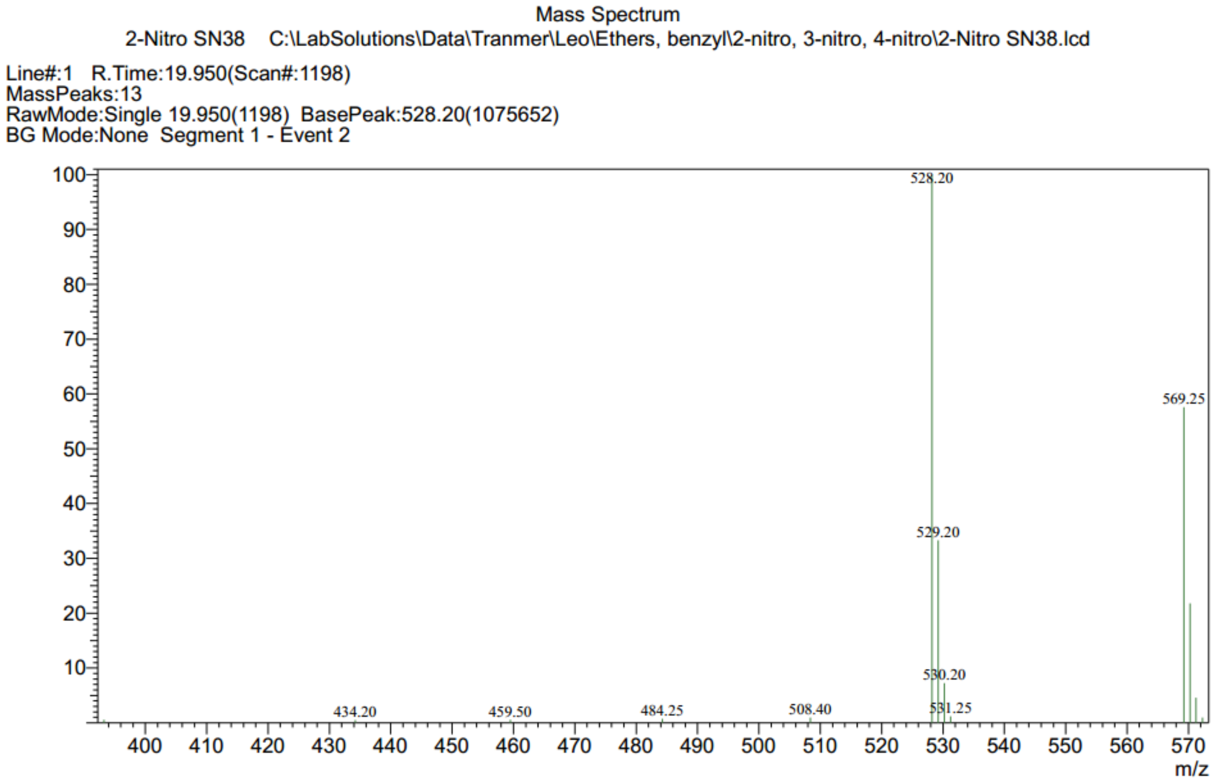

4. Synthesis and Characterization of 3-Nitrobenzyl-SN-38 (3)

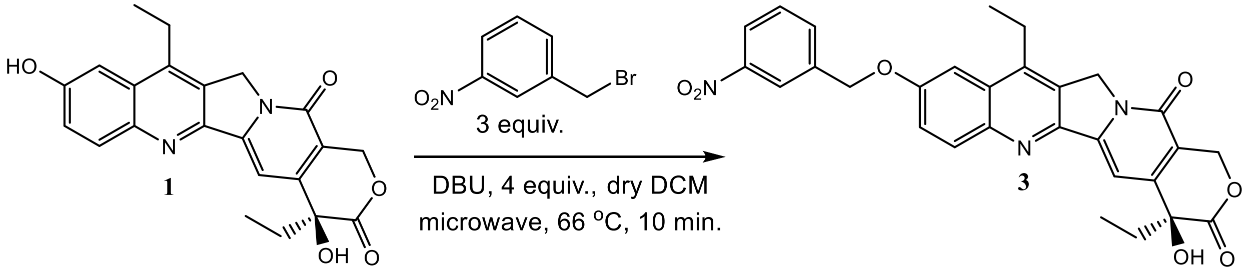

This synthesis was conducted on a microwave synthesizer (Discover®SP W/Activent, CEM, USA). To a microwave reaction vessel (10 mL) were added SN-38 (**1**) (0.0241 g, 0.061 mmol), dry dichloromethane (DCM, 4 mL, dried over molecular sieves overnight), DBU (36 µL, 0.24 mmol) and 3-nitrobenzyl bromide (0.0375 g, 0.17 mmol). The reaction vessel was then sealed and placed in the microwave synthesizer. The reaction was conducted under dynamic mode, 66 ^{o}C, PowerMax mode (simultaneous air cooling, model of microwave synthesizer: Discover®SP W/Activent, CEM, USA), max power 200 W, max pressure 300 psi, for 10 min. The reaction mixture was then purified with silica gel column chromatography on a CombiFlash® Rf 200 purification system, Teledyne Isco, USA, with ethyl acetate in hexane from 0% to 100% (3-nitrobenzyl SN-38 was eluted out with 100% ethyl acetate). Residual solvent was evaporated under vacuum to give 3-nitrobenzyl SN-38 as a yellow powder (0.0216 g, 67%). ^{1}H NMR (400 MHz, DMSO) δ 8.46 (s, 1H), 8.24 (dd, *J* = 8.2, 1.5 Hz, 1H), 8.17 – 8.08 (m, 1H), 8.04 (d, *J* = 7.8 Hz, 1H), 7.75 (t, *J* = 7.9 Hz, 1H), 7.64 (dd, *J* = 6.7, 3.1 Hz, 2H), 7.28 (s, 1H), 6.53 (s, 1H), 5.55 (s, 2H),

5.43 (s, 2H), 5.31 (s, 2H), 3.20 (q, *J* = 7.3 Hz, 2H), 1.96 – 1.77 (m, 2H), 1.24 (t, *J* = 7.6 Hz, 3H), 0.88 (t, *J* = 7.3 Hz, 3H). ^{13}C NMR (101 MHz, DMSO) δ 173.00, 157.27, 157.19, 150.50, 150.27, 148.33, 146.67, 145.00, 144.40, 139.54, 134.75, 132.05, 130.66, 128.89, 128.15, 123.37, 122.96, 122.79, 118.78, 104.35, 96.54, 72.86, 68.88, 65.71, 49.97, 30.69, 22.67, 13.85, 8.23. ESI-MS(+) *m/z* (% relative intensity, [ion]): 528.20 (100, [M + H]$^+$), 569,30 (69.21, [M + H + CH_3CN]$^+$).

4.1 ^{1}H NMR, ^{13}C NMR, and MS spectra of 3-nitrobenzyl-SN-38(3)

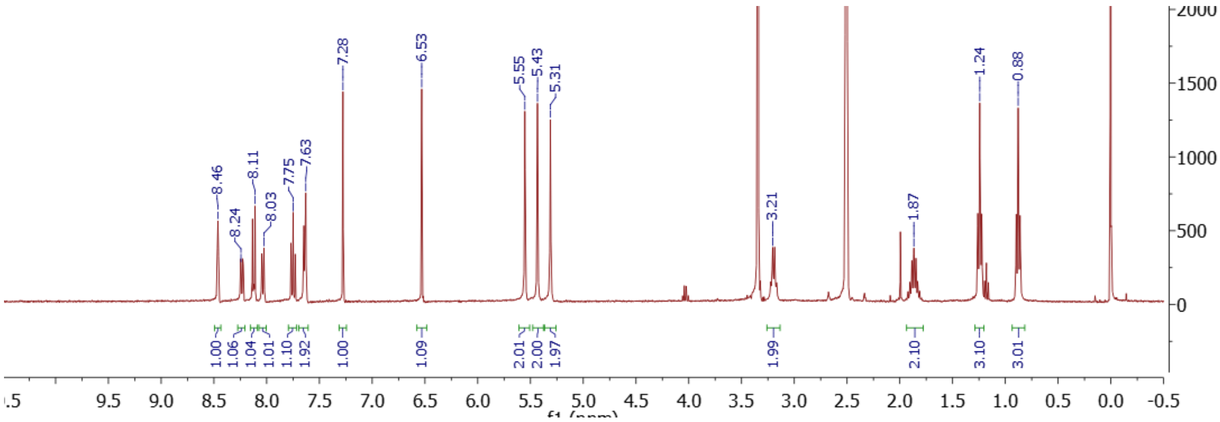

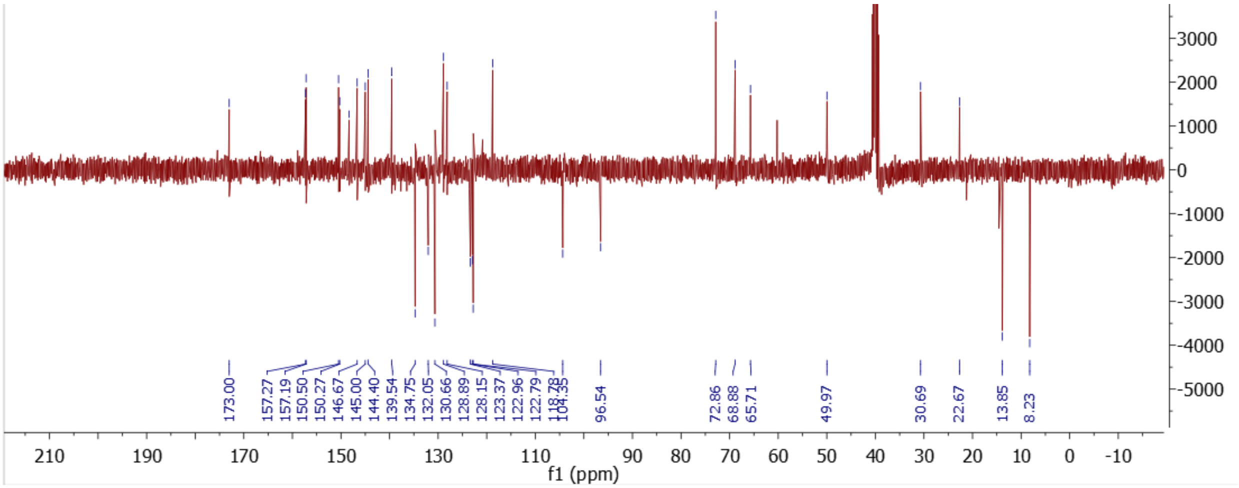

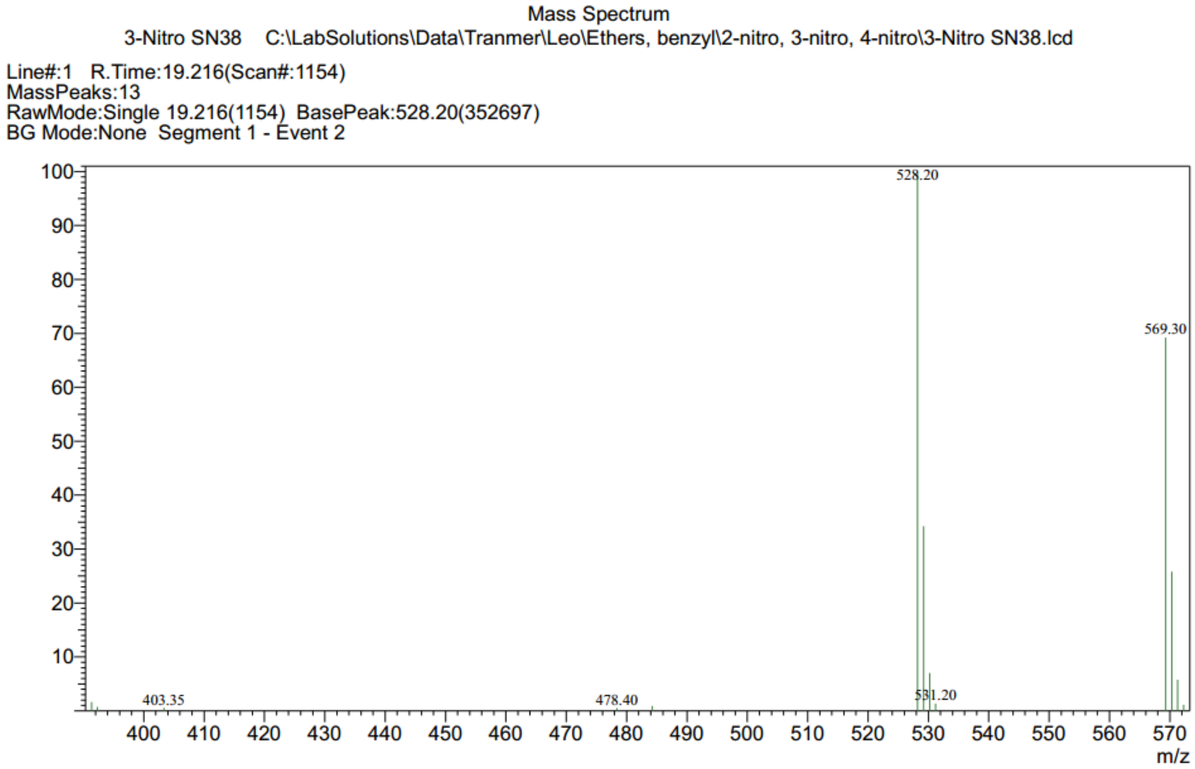

5. Synthesis and Characterization of 4-Nitrobenzyl-SN-38 (4)

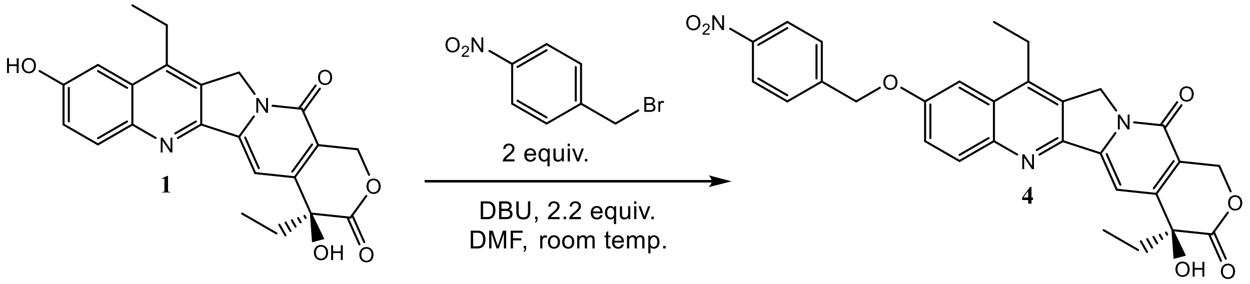

SN-38 (**1**) (0.0936 g, 0.24 mmol) was added into a round bottom flask (10 mL) with a magnetic stirring bar. Dimethylformamide (DMF, 2.0 mL, anhydrous, Sigma-Aldrich) and DBU (80 µL, 0.54 mmol) were then added, and the mixture was sonicated until SN-38 was dissolved. The dissolved SN-38 was then stirred on a stirring plate and purged with argon flow for 20 min. 4-Nitrobenzyl bromide (0.1187 g, 0.55 mmol) was dissolved in 0.75 mL anhydrous DMF and then added slowly (over 30 min) into SN-38 with a syringe. The mixture was allowed to stir at room temperature (22 ^{o}C) for 6 hours. The resulted yellow solid was separated by centrifuging and washed with acetone (2 mL x 5). Residual acetone was then evaporated under vacuum to give 4-nitrobenzyl SN-38 as a yellow powder (0.0864 g, 68%). ^{1}H NMR (400 MHz, DMSO) δ 8.35 – 8.26 (m, 2H), 8.13 (d, *J* = 9.1 Hz, 1H), 7.85 (d, *J* = 8.8 Hz, 2H), 7.63 (dt, *J* = 5.7, 2.6 Hz, 2H), 7.28 (s, 1H), 6.53 (s, 1H), 5.56 (s, 2H), 5.43 (s, 2H), 5.31 (s, 2H), 3.18 (q, *J* = 7.4 Hz, 2H), 1.87 (m, *J* = 14.0, 7.1 Hz, 2H), 1.25 (t, *J* = 7.6 Hz, 3H), 0.88 (t, *J* = 7.3 Hz, 3H). ^{13}C NMR (101 MHz, DMSO) δ 172.98, 157.33, 157.25, 150.54, 150.39, 147.61, 146.72, 145.10, 145.08, 144.49, 132.12, 128.98, 128.23,

124.16, 122.94, 118.84, 104.55, 96.56, 72.87, 71.45, 69.07, 65.74, 50.00, 30.76, 22.68, 13.89, 8.21. ESI-MS(+) *m/z* (% relative intensity, [ion]): 528.25 (100, $[M + H]^+$), 569,30 (92.39, $[M + H + CH_3CN]^+$).

5.1 ^{1}H NMR, ^{13}C NMR, and MS spectra of 4-nitrobenzyl-SN-38 (4)

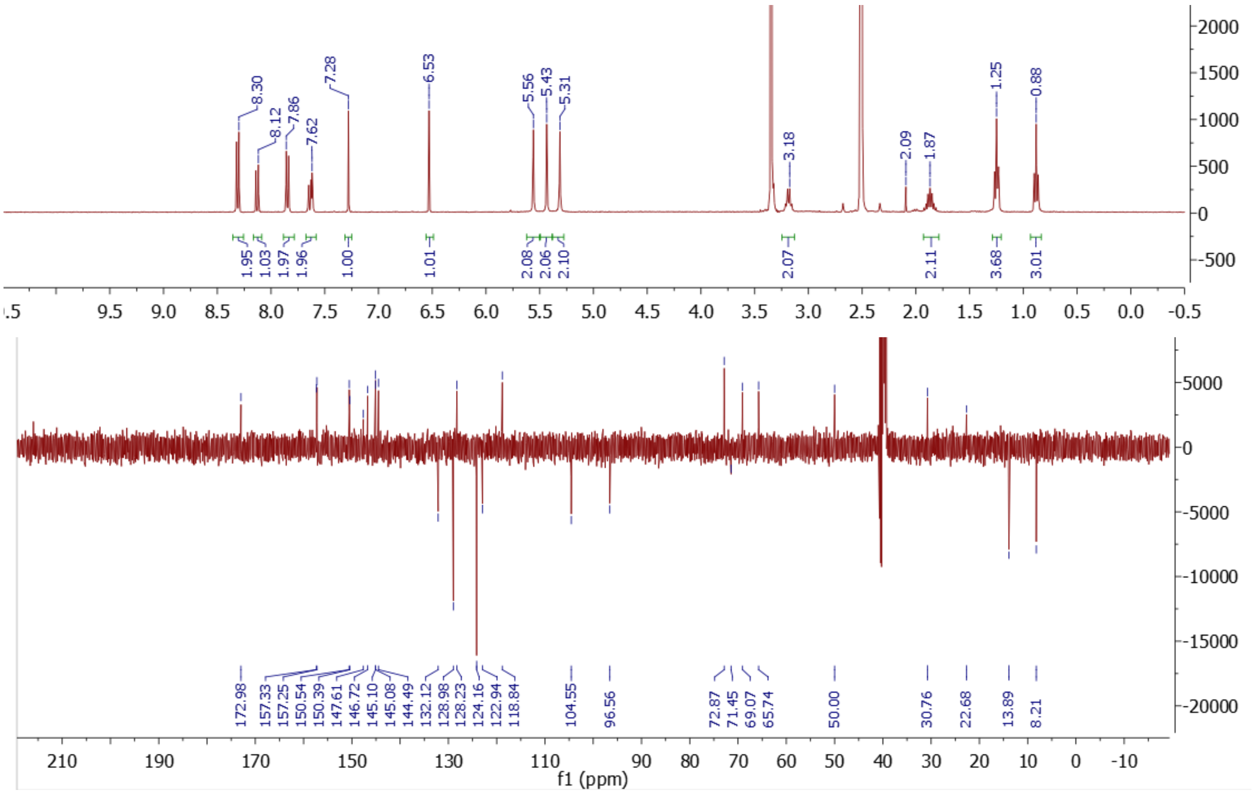

Mass Spectrum

4-Nitro SN38   C:\LabSolutions\Data\Tranmer\Leo\Ethers, benzyl\2-nitro, 3-nitro, 4-nitro\4-Nitro SN38.lcd

Line#:1   R.Time:19.583(Scan#:1176)
MassPeaks:14
RawMode:Single 19.583(1176) BasePeak:528.25(511751)
BG Mode:None Segment 1 - Event 2

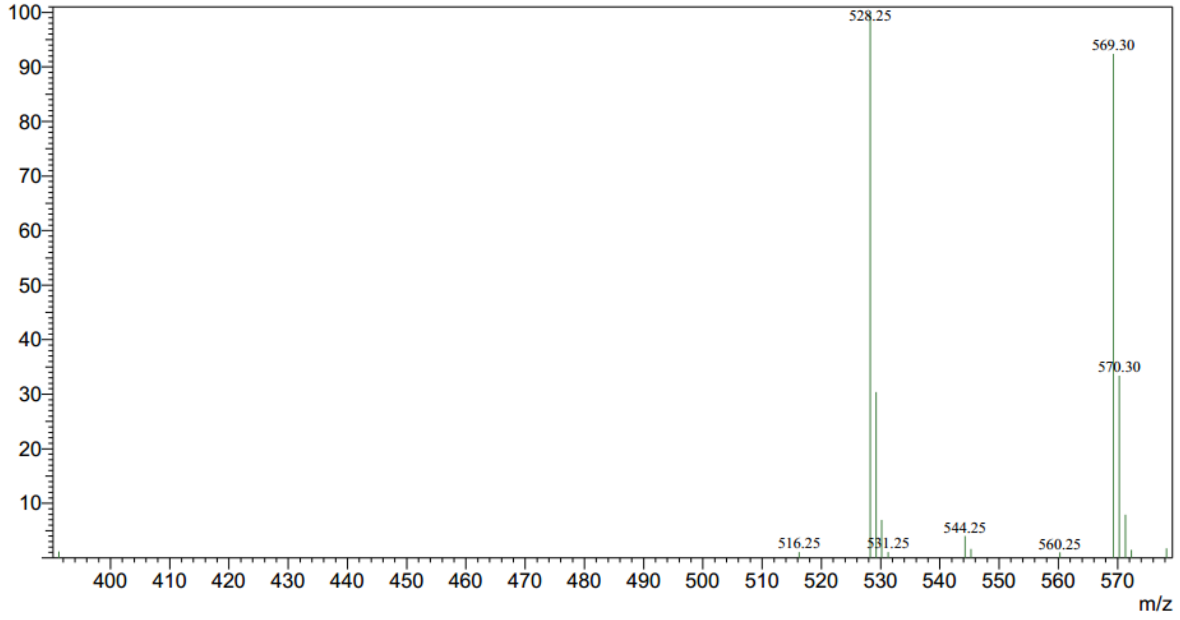

A. Retention time and MS spectra for SN-38 (**1**), approximately 13.6 min.
  - *SN-38 (**1**)*; 393 $[M + H]^+$, 434 $[M + H + CH_3CN]^+$.

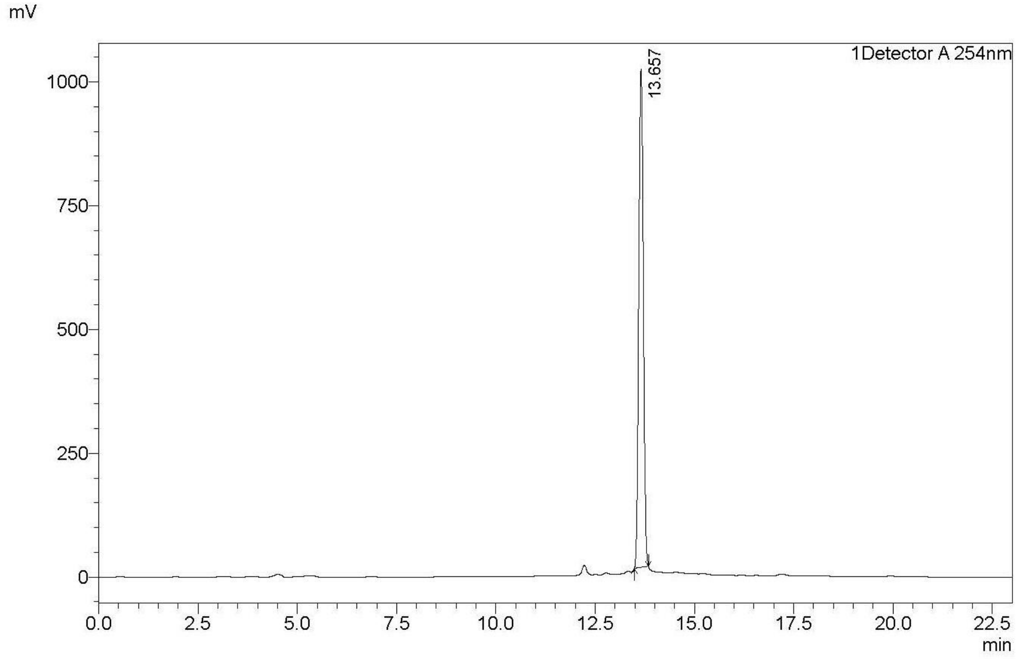

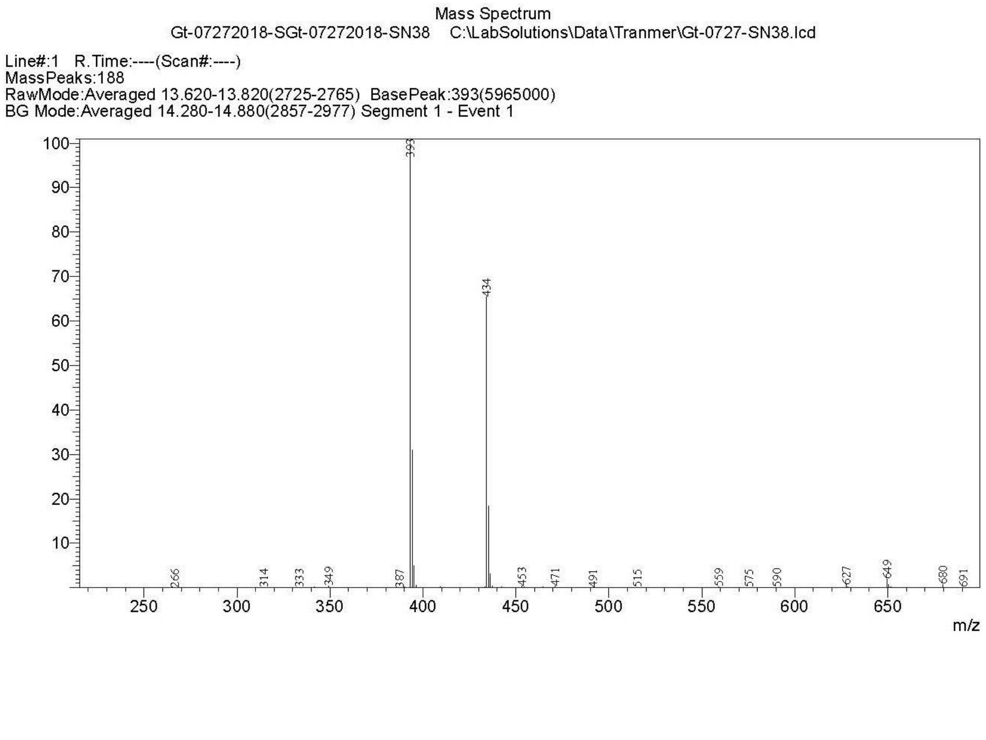

B. Retention time and MS spectra for 4-Nitro Analog (**4**), approximately 17.3 min.

- *4-Nitrobenzyl-C_{10}-SN-38 (**4**)*; 528 $[M + H]^+$, 569 $[M + H + CH_3CN]^+$.

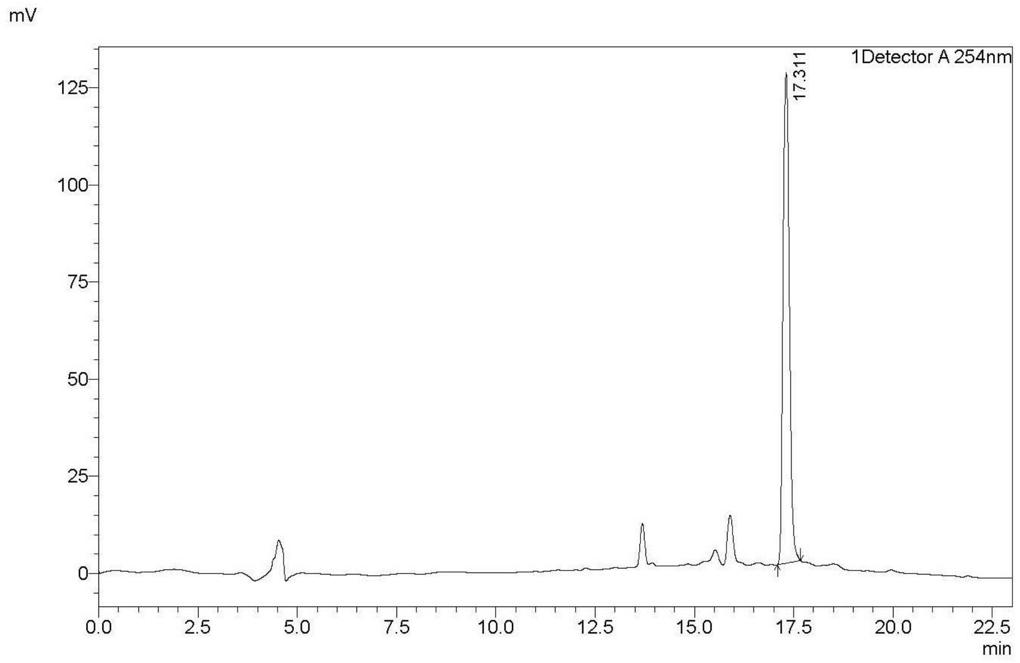

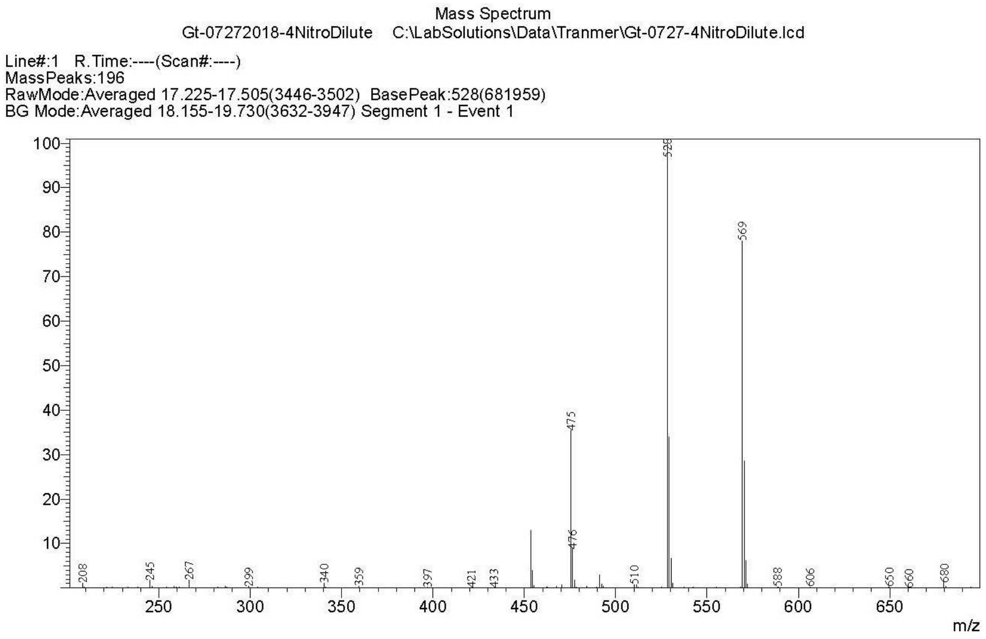

C. LC-MS of crude reaction of **4** with Zn dust and AcOH after 10 minutes.

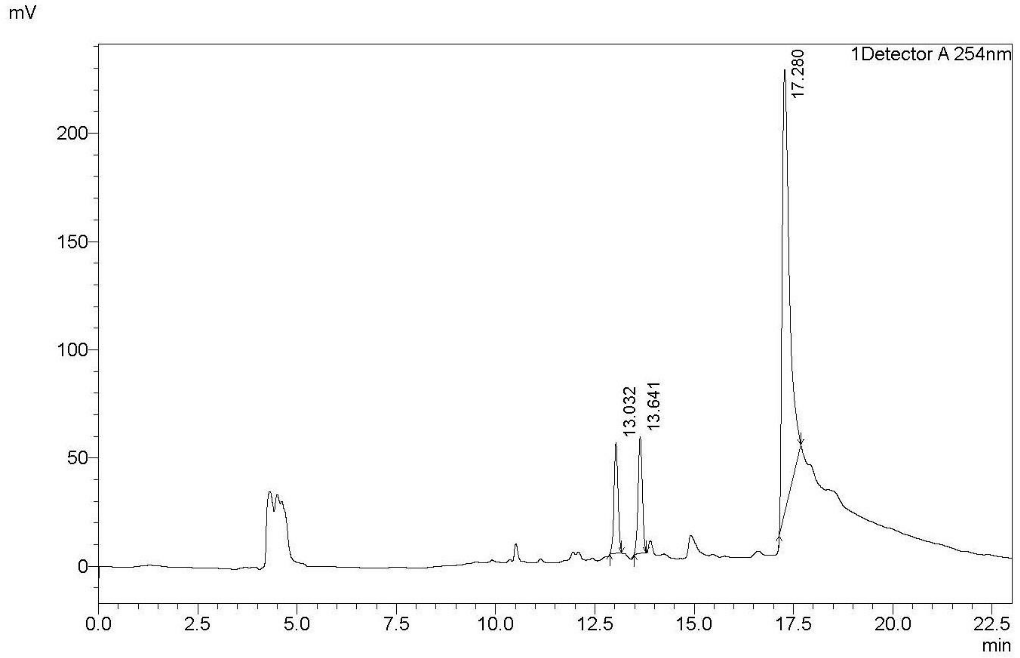

D. LC-MS of crude reaction of **4** with Zn dust and AcOH after 8 hours.

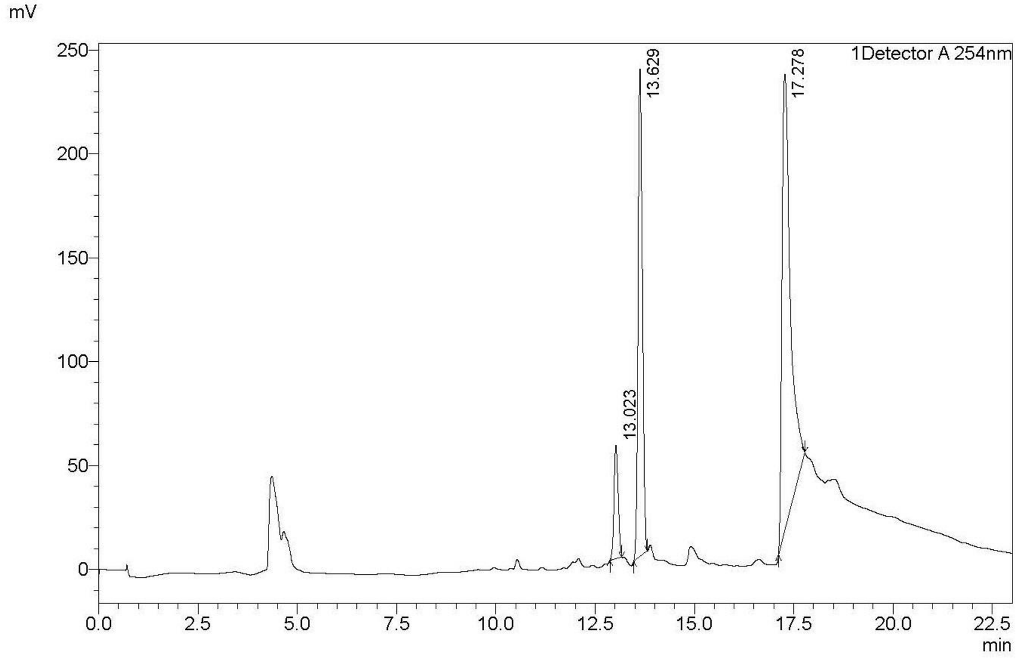

E. Mass spectrum for 13.023 minute peak for 8 hour reaction (reduced form of SN-38)
   - *'Reduced' SN-38 (+2 hydrogens)*; 395 $[M + H]^+$, 434 $[M + H + CH_3CN]^+$.

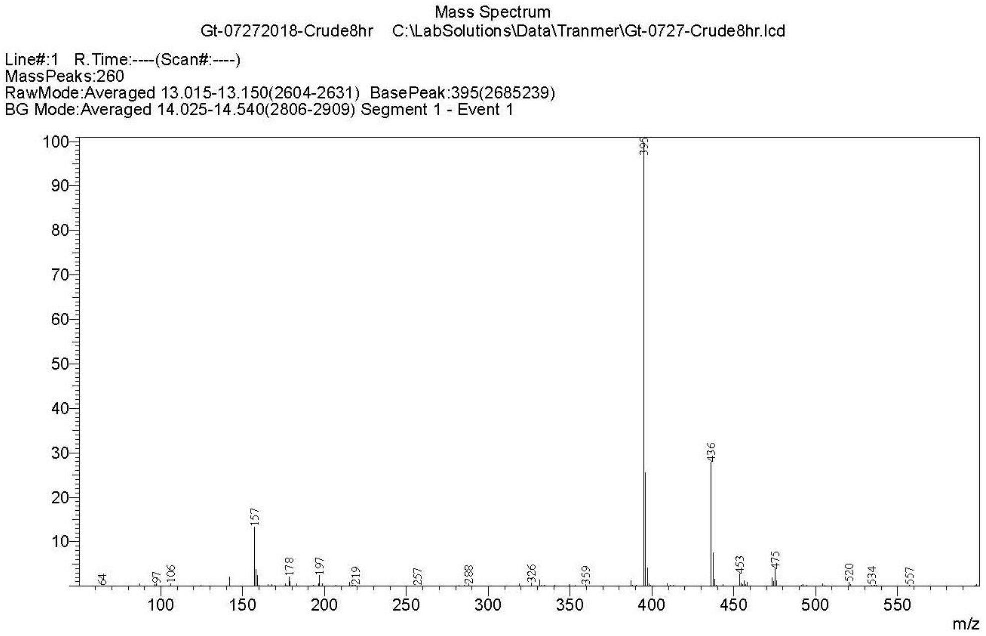

F. Mass spectrum for 13.629 minute peak for 8 hour reaction (SN-38 (**1**))
   - *SN-38 (**1**)*; 393 $[M + H]^+$, 434 $[M + H + CH_3CN]^+$.

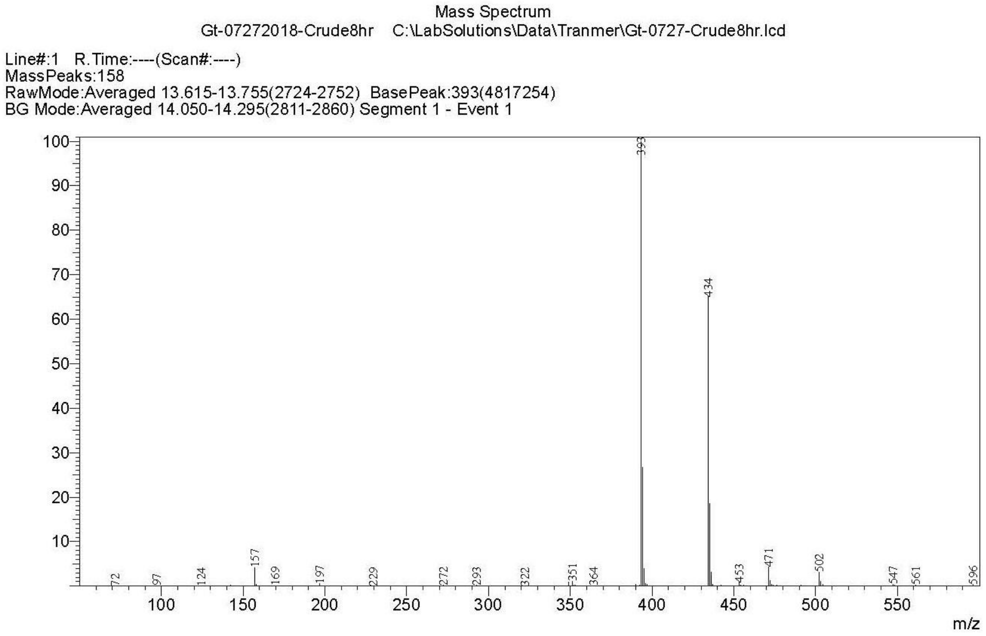

Supplement: Supplementary file 1 [file molecules-23-02041-s001.pdf]
